# Large-scale interaction effects reveal missing heritability in schizophrenia, bipolar disorder and posttraumatic stress disorder

**DOI:** 10.1038/tp.2017.61

**Published:** 2017-04-11

**Authors:** H J Woo, C Yu, K Kumar, J Reifman

**Affiliations:** 1 Biotechnology High Performance Computing Software Applications Institute, Telemedicine and Advanced Technology Research Center, U.S. Army Medical Research and Materiel Command, Fort Detrick, MD, USA

## Abstract

Genetic susceptibility factors behind psychiatric disorders typically contribute small effects individually. A possible explanation for the missing heritability is that the effects of common variants are not only polygenic but also non-additive, appearing only when interactions within large groups are taken into account. Here, we tested this hypothesis for schizophrenia (SZ) and bipolar disorder (BP) disease risks, and identified genetic factors shared with posttraumatic stress disorder (PTSD). When considered independently, few single-nucleotide polymorphisms (SNPs) reached genome-wide significance. In contrast, when SNPs were selected in groups (containing up to thousands each) and the collective effects of all interactions were estimated, the association strength for SZ/BP rose dramatically with a combined sample size of 7187 cases and 8309 controls. We identified a large number of genes and pathways whose association was significant only when interaction effects were included. The gene with highest association was *CSMD1*, which encodes a negative regulator of complement activation. Pathways for glycosaminoglycan (GAG) synthesis exhibited strong association in multiple contexts. Taken together, highly associated pathways suggested a pathogenesis mechanism where maternal immune activation causes disruption of neurogenesis (compounded by impaired cell cycle, DNA repair and neuronal migration) and deficits in cortical interneurons, leading to symptoms triggered by synaptic pruning. Increased risks arise from GAG deficiencies causing complement activation and excessive microglial action. Analysis of PTSD data sets suggested an etiology common to SZ/BP: interneuron deficiency can also lead to impaired control of fear responses triggered by trauma. We additionally found PTSD risk factors affecting synaptic plasticity and fatty acid signaling, consistent with the fear extinction model. Our results suggest that much of the missing heritability of psychiatric disorders resides in non-additive interaction effects.

## Introduction

Schizophrenia (SZ) and bipolar disorder (BP) are severe psychiatric disorders with overlapping symptoms affecting ~1% of the population.^1, 2^ Although evidence points to strong genetic risks,^3^ identification of the underlying genetic factors has been challenging because of high polygenicity, necessitating large sample sizes in meta-analyses.^4^ Possible ways to take polygenicity into account include combining single-nucleotide polymorphisms (SNPs) into groups, for example, based on genes or pathways, and using methods such as enrichment analysis^5, 6^ or aggregated tests.^7, 8^ However, these methods still do not include non-additive interaction effects. Extensions of the independent loci (IL) analysis by pairwise testing may partially capture such effects, but in the largest SZ meta-analysis to date, it was reported that pairwise tests did not yield new associations.^4^ In this work, we characterized non-additive interaction effects of common variants on SZ and BP risks using a recently developed algorithm (discrete discriminant analysis, DDA),^9, 10^ which greatly extends the scale of interacting partners simultaneously considered beyond SNP pairs by using the regularized inference of high-dimensional interactions within large SNP groups. Over-fitting is avoided by optimizing the risk prediction quantified by the area under the curve (AUC) of receiver operating characteristics, estimated from cross-validation. We refer to such optimized inference of the aggregated sum of all possible interaction effects as the collective loci inference.

Using moderately sized collections of SZ and BP case–control samples, we first found that standard IL analyses without interaction could detect only marginal levels of association. In contrast, inclusion of interaction effects led to the emergence of a large number of highly significant SNP groups. These collectively interacting genetic factors (genes and pathways) comprised not only loci with documented genetic association but also varied arrays of neurodevelopmental pathways, whose roles in SZ/BP phenotypes have so far been inferred largely through evidence from non-genetic studies. In particular, the pathway groups collectively point to the insufficient development of cortical inhibitory interneurons^11^ as the main cause underlying the disease phenotypes.

We further supported this main conclusion by analyzing posttraumatic stress disorder (PTSD) data sets. PTSD is characterized by the inability to recover from trauma-induced symptoms, including flashback and avoidance.^12, 13^ Recent genome-wide association studies identified potential risk loci,^14, 15, 16, 17, 18^ but connections to fear conditioning/extinction, a powerful model for PTSD,^19^ are yet to be established. We analyzed two PTSD data sets using our algorithm and found a significant overlap of associated neurodevelopmental pathways with SZ/BP results, which provides an explanation for the observed comorbidity of these phenotypes.^20, 21, 22^ Our results further suggest significant association of pathways central to fear conditioning/extinction, including AMPA receptor trafficking, γ-aminobutyric acid (GABA) synthesis and degradation, and fatty acid-regulated nociceptive signaling.

## Materials and methods

### Genotype data

For SZ, we used Molecular Genetics of Schizophrenia Genetic Association Information Network (GAIN) (dbGaP accession number phs000021.v3.p2) and non-GAIN studies (phs000167.v1.p1) data.^23^ We used the quality control-filtered GAIN data for autosomal chromosomes. For the non-GAIN sample, we applied minor allele frequency >0.01, call rate >95% and Hardy–Weinberg (HW) equilibrium *P*>10^−6^ filters to get 714 608 SNPs common to European American (EA) and African American (AA) groups. We then combined GAIN and non-GAIN samples for EA and AA groups separately (Supplementary Table 1) and selected 670 557 SNPs common to both groups. For BP, we applied the same filters to GAIN data (phs000017.v3.p1)^24^ to obtain 676 829 autosomal SNPs common to EA and AA. We additionally used Wellcome Trust Case Control Consortium (WT) BP data and followed quality control steps as described:^25^ we first identified SNPs with independent-SNP *P*-value *P*_i_<10^−3^ and removed variants with poor clustering in call signals. We further removed SNPs with high apparent association in isolation to obtain 458 846 SNPs in total. We then identified 343 070 SNPs common to GAIN and WT samples for further analysis (Supplementary Table 1). We treated GAIN EA, AA and WT groups as three separate subpopulations in our analyses. For SZ+BP sample, we used 337 388 SNPs shared by SZ and BP data. Five sub-samples (SZ EA/AA, BP EA/AA/WT) containing this common SNP set were prepared for SZ+BP analysis.

We formed PTSD case and control groups from the alcohol, cocaine and opioid dependence (ACOD) data set^14, 26^ by selecting trauma-exposed subjects with known PTSD status, divided into EA and AA groups based on the stratification information of the original data (818 cases and 2605 controls; see Supplementary Table 1). We only included non-imputed autosomal SNPs (695 308 in total) and applied the quality control filter of minor allele frequency >0.01 and HW *P*>10^−6^ to obtain 634 281 SNPs. We further used the Marine Resilience Study (MRS) data set^16^ (phs000864.v1.p1), selecting subjects meeting DSM-IV criteria A1 (524 cases and 3304 controls). We used principal component analysis along with self-reported ethnicity to stratify subjects into EA, AA and AS (Asian/others) groups (Supplementary Table 1). We selected SNPs in the dbSNP database and used call rate >0.95 and HW *P*-value >10^−6^ to obtain 842 950 SNPs. We also formed a combined data set (ACOD+MRS) by selecting 443 198 SNPs shared by both data sets, containing five distinct subgroups (ACOD EA/AA, MRS EA/AA/AS).

### Meta-analysis

We extended the DDA algorithm^9^ to account for population heterogeneity, similar in spirit to other meta-analysis algorithms^27, 28^ except that it is also applicable to interacting SNPs (Supplementary Text). Except noted otherwise, we used the genotypic model throughout this work. We used two-, three- and five-sample versions for SZ, BP and SZ+BP data sets, respectively. The SZ and BP EA/AA control groups shared 1676 individuals (Supplementary Table 1); since individual-level genotype information is used only within each subsample during inference, this overlap does not affect the combined statistics, and the sample size quoted treated the control groups as distinct. We used fivefold cross validations to maximize AUC with respect to *ɛ*. We observed that any degree of SNP filtering based on whole-sample IL *P*-value before cross-validation led to a severe inflation of AUCs and refrained from global quality control filtering based on the *P*-values.

### Pathway/gene-based SNP selection

**Figure 1:**
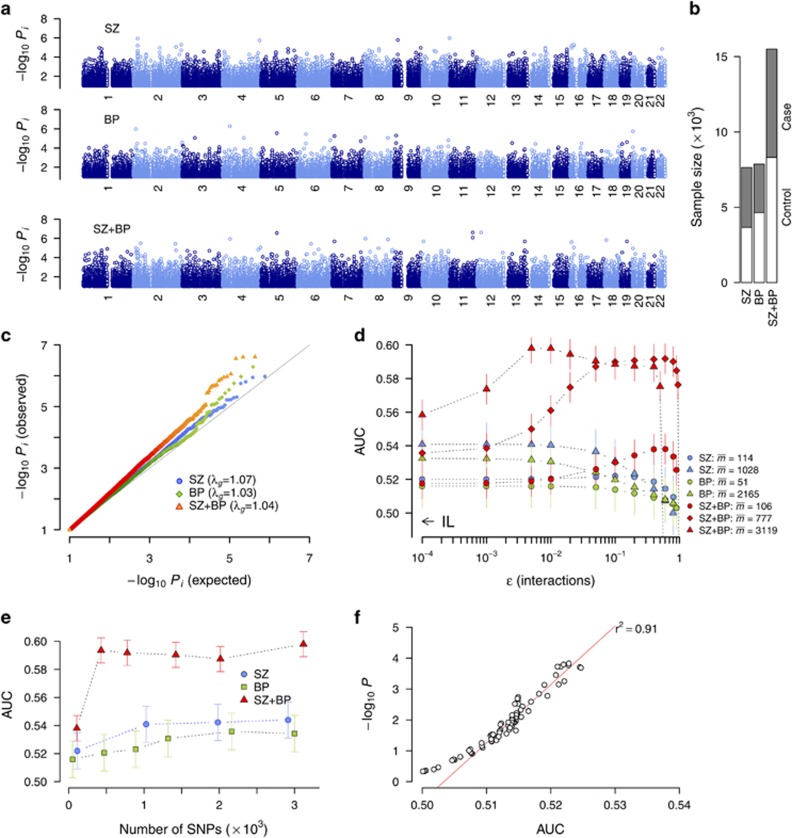
Disease association in independent loci and collective interaction analyses. (**a**) Independent loci (IL) *P*-value *P*_i_ for schizophrenia (SZ), bipolar disorder (BP) and SZ+BP data. (**b**) Sample sizes (see Supplementary Table 1). (**c**) Quantile–quantile plots and inflation factor *λ*_g_. (**d**) Variations in the area under curve (AUC) with interaction strength *ɛ*. Single-nucleotide polymorphisms (SNPs) were selected based on *P*_i_ during cross-validation. The mean number of SNPs are shown in legend. (**e**) Dependence of maximum AUC on mean number of SNPs. (**f**) Correspondence between AUC and *P*-value of SNP sets. The red line is linear regression for AUC >0.51. Error bars and vertical lines, 95% confidence interval.

We used 1730 Reactome pathways^29^ downloaded from http://www.reactome.org on 9 April 2016. To each pathway, we assigned the union of all SNPs within 50 kb of the coding region of the gene set. For collective loci inference with *m*~10 000 or more, we combined SNP selection with cross-validation based on *P*_i_ (IL *P*-value). The AUC versus *P*-value relationship (Figure 1f) was obtained by permutation using a selection of pathways with AUC as the statistic. For gene-based groups, we selected SNPs within 50 kb of the coding regions of 18 338 genes. Scaling of computational costs with increasing SNP numbers is shown in Supplementary Figure 1: inferences with less than ~1000 SNPs can be completed without difficulty on desktop machines. Larger pathways require a parallel architecture.

### Code availability

The updated version of the software GeDI (genotype distribution-based inference) is available at http://www.github.com/BHSAI/GeDI.

## Results

### Disease association without interaction effects

Our SZ analysis was based on Molecular Genetics of Schizophrenia data set^23^ comprising EA and AA subpopulations with 3971 cases and 3666 controls in total. For BP, we combined the GAIN samples (EA and AA)^24^ with WT data^25^ (3216 cases and 4643 controls in total; Supplementary Table 1). We first characterized the level of association achievable under IL using the special case of DDA with interactions turned off:^9^ with each SNP tested separately, these data sets in this special case are expected to yield results closely matching results from the original reports. When analyzed separately, the sub-samples (EA/AA for SZ and EA/AA/WT for BP) each had weak levels of association (Supplementary Figure 2). The multi-sample extension of DDA efficiently corrected for inflation from population structures. The SZ loci with strongest association were consistent with the original report (Supplementary Figure 3).^23^ Both SZ and BP meta-analysis results showed low levels of association (Figure 1a). We compared our SZ/BP IL *P*-value profiles with the available summary statistics from the Psychiatric Genomics Consortium (PGC) studies.^4, 30^ The Molecular Genetics of Schizophrenia and GAIN sub-samples used in our study are contained in the PGC studies, which, however, are much larger in combined sample sizes. For SZ, the association levels of a number of loci showed considerable overlap with those in the PGC (Supplementary Figure 4). For BP, within some of PGC-identified loci, groups of SNPs with IL *P*-value *P*_i_ down to ~10^−3^ could be located within our BP results (Supplementary Figure 5), suggesting that although relatively small in size, our data sets are representative of typical samples recruited into large-scale meta-analyses, contributing a few but not all of IL genomic features. We also considered the data set combining SZ and BP samples (SZ+BP; Figure 1b) comprising five subpopulations, with which we observed a moderate increase in the number of SNPs with *P*_i_ ranging from 10^−2^ to 10^−5^ (Figure 1c).

### Collective interaction effects among SNPs selected genome-wide

We next sought to characterize how interaction effects modify the IL results. The choice of models rests solely on the selection of SNPs from genome-wide data apart from the choice of genotypic, dominant or recessive model. We built statistical models of varying sizes by selecting SNPs genome-wide based on their IL association strengths during cross-validation using a cutoff *P*_i_ value: with the sample divided into training and test sets of 4:1 ratio, we derived IL *P*-value *P*_i_ of all genome-wide SNPs based on the training set, and selected SNPs with *P*_i_ less than the cutoff for inclusion in the model. We then inferred all interaction effects involving these SNPs using the training set, derived the disease risk and assessed the prediction performance for the test set via AUC. This procedure was repeated for different values of regularization parameter *ɛ*. Its value *ɛ*=0 corresponds to the IL limit, at which the AUC ranged from 0.52 to 0.54 for SZ and BP (Figure 1d). The other limit of *ɛ*=1 corresponds to the strongly interacting limit, where over-fitting is seen to result in a sharp drop in AUC. The maximization of the AUC under cross-validation allows us to avoid over-fitting and estimate the relative goodness-of-fit, which we directly verified by emulating our inference conditions using simulated data (Supplementary Figure 6). For SZ and BP, AUC improved only marginally when *ɛ* was increased from 0 (Figure 1d) for SNP sets of sizes up to ~3000 (Figure 1e). In contrast, for the combined SZ+BP data, AUC rapidly rose with increasing *ɛ* near the IL limit (Figure 1d). The maximum AUC increased significantly with increasing number of SNPs *m* before saturating at ~0.59 for *m* larger than ~1000 (Figure 1e). We estimated the relationship between AUC and *P*-value (representing the significance of the interacting SNP group) with permutation resampling and found −log_10_ *P* to be highly linear for AUC >0.51 (*r*^2^=0.9; Figure 1f). In this procedure, the AUC calculation was repeated many times with randomly shuffled data and the *P*-value was estimated as the fraction of times where the AUC under randomized data was larger than for the original data. The predicted relationship yielded *P*-value estimates of *P*~10^−7^, 10^−15^ and 10^−18^ for AUC=0.54, 0.58 and 0.60, respectively. Based on this estimate, the Bonferroni-corrected type I error rate <0.05 threshold among 2000 groups of SNPs (for example, pathways) is *P*<2.5 × 10^−5^ or AUC >0.53 because significance tests were performed once for each pathway (see Supplementary Table 2). We thus conclude that the maximum AUC levels reached in SZ and BP data (0.54 (0.01) and 0.53 (0.01), respectively; 95% confidence interval in parentheses; Figure 1e) are close to but above stringent thresholds for discovery, whereas inclusion of interactions in SZ+BP data led to a dramatic increase in significance levels.

### Collective interaction effects within genes and pathways

We used Reactome database^29^ and constructed pathway-based SNP sets. For comparison, we first analyzed the three data sets using gene set enrichment-based method MAGENTA.^31^ For SZ+BP, the lowest false discovery rate and enrichment *P*-value were 0.46 and 4.0 × 10^−4^, respectively (75 percentile gene score cutoff). SZ and BP outcomes were similar (Supplementary Figure 7). The top significance levels of pathways (equivalent to AUC =0.525 from Figure 1f) were thus comparable to our IL results (*ɛ*=0 limit in Figure 1d). A recent PGC meta-analysis of ~60 000 individuals used enrichment-based pathway analyses including MAGENTA, and showed that *P*-values down to ~10^−6^ could be achieved without interaction effects.^6^

**Figure 2:**
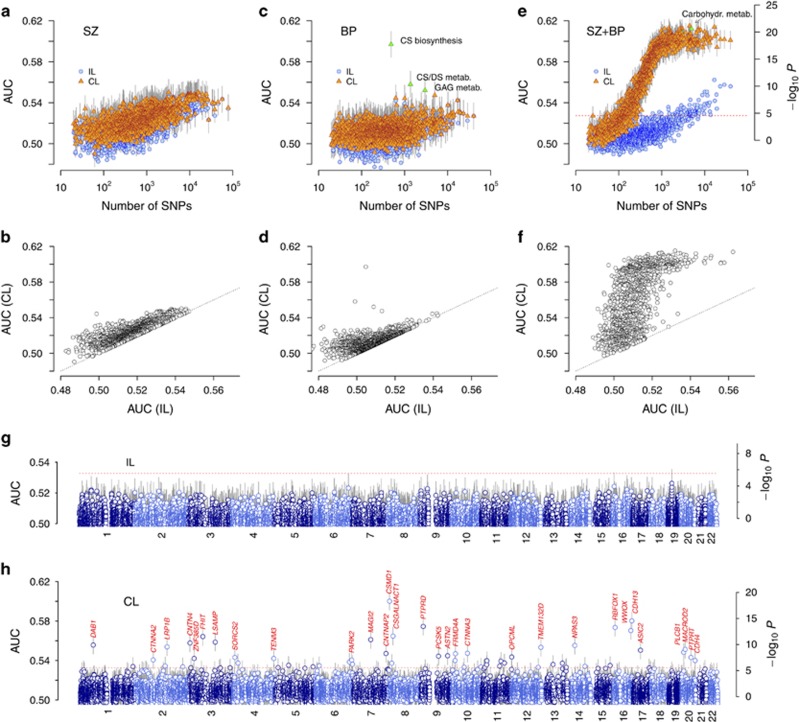
Distribution of pathway and gene association strengths. (**a**–**f**) Pathway-based results for SZ (**a** and **b**), BP (**c** and **d**) and SZ+BP (**e** and **f**) data sets. Each pathway was scored by AUC with no interaction (*ɛ*=0; IL) and with *ɛ*-optimized interactions (CL). Green symbols in **c** and **e** are CL results for annotated pathways. (**g** and **h**) Gene-based results for SZ+BP without (**g**) and with interactions (**h**). Symbols for each gene are located at the midpoint of coding regions in corresponding chromosomes. See Table 1 and Supplementary Table 4. The *P*-value estimates are based on Figure 1f. Dotted lines represent the Bonferroni thresholds. Vertical lines, 95% confidence interval. AUC, area under the curve; BP, bipolar disorder; Carbohydr., carbohydrate; CL, collective loci; CS, chondroitin sulfate; DS, dermatan sulfate; IL, independent loci; metab., metabolism; SZ, schizophrenia.

We next evaluated each pathway using collective inference. The qualitative trend in the distribution of pathway size versus AUC (Figures 2a, c and e) closely matched Figure 1e: in both SZ and BP, AUC moderately increased with increasing *m*, with AUC in SZ results slightly higher than in BP. Inclusion of interactions improved AUC moderately (Figures 2b and d) in both cases. Notable exceptions were the two pathways ranked highest in BP: Chondroitin sulfate (CS) biosynthesis (AUC=0.597 (0.013)) and CS/dermatan sulfate metabolism (AUC=0.558 (0.013)), strongly suggesting the key role of CS synthesis in BP. The rank orders for pathways in SZ and BP results were significantly correlated (Kendall's *τ*=0.15, *P*~10^−20^), indicating common disease mechanisms. In contrast to SZ and BP data sets, we observed a strong increase in association strength with the inclusion of interactions in SZ+BP (Figures 2e and f) as in Figures 1d and e. Pathways with more than 1000 SNPs had median AUC of 0.60 and 0.52 with and without interactions, respectively (see Supplementary Table 3 for the full list). The highest-ranked pathways included Class A/1 (rhodopsin-like receptors; AUC=0.615 (0.009), *P*~10^−21^) of the signaling by G-protein-coupled receptor group and metabolism of carbohydrates (AUC=0.612 (0.009), *P*~10^−21^), the latter again suggesting the importance of proteoglycans. We used two representative pathways of different sizes to examine the performance of collective inference with varying sample sizes, level of linkage disequilibrium and models (Supplementary Figure 8). The location of maxima in AUC with respect to *ɛ* shifted to lower values with larger *m*. Down-sampling of SZ+BP data set clearly showed that the rise in association strength was from the increase in sample size compared with SZ and BP, and suggested that the current sample size was far from saturation (Supplementary Figure 8b). We observed that linkage disequilibrium-based pruning of SNPs in each pathway either increased or did not affect the AUC values down to *r*^2^ maximum of ~0.1, which reduced the number of SNPs to ~50% or less, and beyond (Supplementary Figure 8c). For these two pathways, the genotypic model fit was better than (or similar to) those with the recessive (dominant) model. We further compared the performance of the dominant, recessive and genotypic models for a subset of pathways and found the genotypic model to consistently provide better fits (Supplementary Figure 8e).

We also considered SNP groups based on genes using SZ+BP data. With 17 821 gene-based SNP sets, the Bonferroni threshold is *P*=2.8 × 10^−6^, or from Figure 1f, AUC=0.533. We scored these SNP groups without (IL) and with interactions (collective loci): whereas all genes had significance levels well below the genome-wide threshold under IL (Figure 2g), with interactions included, ~50 genes had association strengths above the threshold (Figure 2h and Supplementary Table 4). The gene with highest association was *CSMD1*, which in many previous studies has been linked to SZ^32, 33, 34, 35^ as well as BP.^36, 37, 38, 39^ Most of the other top-ranked genes also had documented roles in neurodevelopment or psychiatric disorders (Table 1). Notably, *CSGALNACT1* codes for an enzyme synthesizing CSPGs, supporting our earlier observation of the high association of GAG synthesis (Figure 2c).

### SZ/BP disease mechanism inferred from pathways

**Figure 3:**
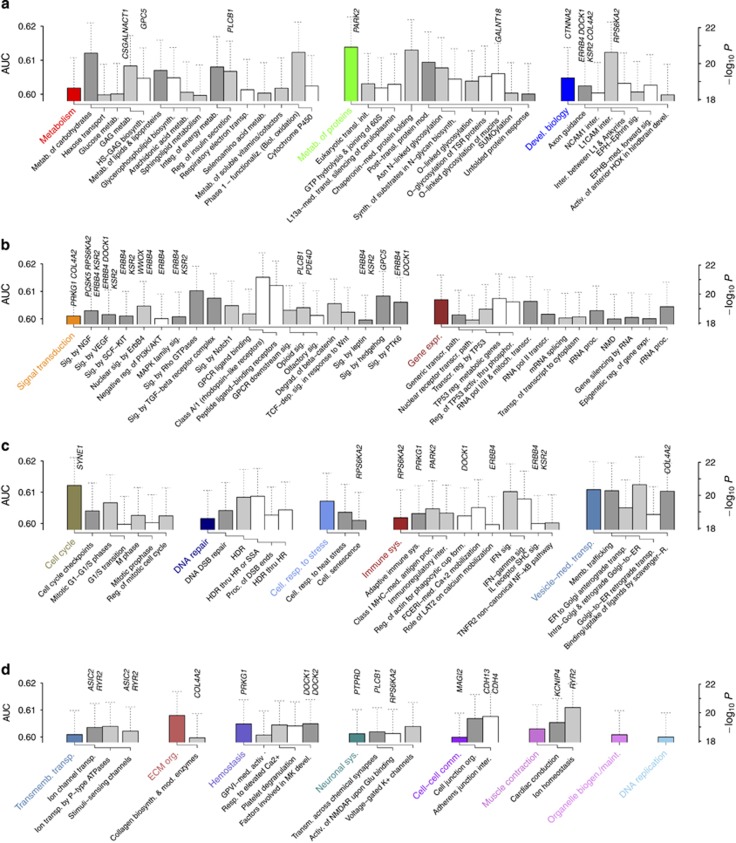
Pathways highly associated with SZ+BP under collective inference. (**a**–**d**) Pathways in Figure 2e with area under the curve (AUC) >0.60 organized into Reactome hierarchy.^29^ See Supplementary Table 3 for full list. Genes of genome-wide significance under collective inference (Figure 2h and Supplementary Table 4) within each pathway are labeled vertically. Error bars, 95% confidence interval. Activ., activation/activates/activated/activity; AJ, adherens junction; Akt, protein kinase B; Asn, asparagine; biogen., biogenesis; biol., biological; biosynth., biosynthesis; BP, bipolar disorder; CAM, cell adhesion molecule; cell., cellular; comm., communication; CS, chondroitin sulfate; degrad., degradation; dep., dependent; devel., development; DS, dermatan sulfate; DSB, double-strand break; ECM, extracellular matrix; EGFR, epidermal growth factor receptor; EPH, erythropoietin-producing hepatocellular carcinoma; Ephrin, EPH receptor interacting proteins; ER, endoplasmic reticulum; ErbB4, erb-b4 receptor tyrosine kinase 4; expr., expression; FCERI, Fc epsilon receptor 1; form., formation; functionaliz., functionalization; GAG, glycosaminoglycan; GPCR, G-protein-coupled receptor; GPVI, glycoprotein VI; GRB, growth factor receptor-bound protein; HDR, homology-directed repair; HOX, homeobox; HR, homologous recombination; HS, heparan sulfate; integ., integration; IFN, interferon; IGF1R, insulin growth factor 1 receptor; IL, interleukin; init., initiation; inter., interaction; LAB, linker for activation of B cells; LAT2, linker for activation of T cells family, member 2; maint., maintenance; MAPK, mitogen-activated protein kinase; med., mediated; memb., membrane; metab., metabolism; mitoch., mitochondrial; MHC, major histocompatibility complex; MK, megakaryocyte; mod., modification/modifying; NCAM, neural CAM; NGF, nerve growth factor; NMD, nonsense-mediated decay; NMDAR, *N*-methyl-d-aspartate receptor; NF-κB, nuclear factor κB; org., organization; PI3K, phosphatidylinositol-3-kinase; phosphor., phosphorylation; pol, polymerase; proc., processing; PTK, protein tyrosine kinase; R., receptor; RAF, rapidly accelerated fibrosarcoma; reg., regulated/regulation; resp., response; SCF, stem cell factor; SHC, Src homology domain-containing; sig., signaling; SLC, solute-carrier; SOS, son of sevenless; SSA, single-strand annealing; SUMO, small ubiquitin-like modifiers; synth., synthesis; sys., system; SZ, schizophrenia; TCF, T cell factor; TGF, transforming growth factor; TNFR, tumor necrosis factor receptor; TP53, tumor protein p53; transcr., transcription; transl., translation; transmemb., transmembrane; transm., transmission; transp., transport; TSR, thrombospondin type 1 repeat domain-containing; thru, through; VEGF, vascular endothelial growth factor.

We used pathways highly associated with SZ+BP to infer pathogenesis mechanisms common to SZ and BP. The five classes of pathways shown in Figures 3a–c suggested a broad overview: neurogenesis (developmental biology) is impaired by molecular risk factors (metabolism of carbohydrates and proteins) regulated by gene expression and signal transduction, leading to excessive immune activation (immune system). Underlying the major SZ symptoms observed in adolescence is the inadequate inhibitory action of GABAergic interneurons.^11, 41^ Abnormalities in neurogenesis,^42^ migration^43, 44^ and synaptic remodeling^45^ are all expected to contribute to the cortical interneuron deficiency. We found possible early risk factors in maternal immune activation^46^ with Fc *ɛ* receptor I-mediated calcium mobilization, signaling by stem cell factor-KIT and negative regulation of phosphoinositide 3-kinase/AKT (Figures 3b and c): allergic reactions during pregnancy cause mast cell activation and microglial activation (Supplementary Figure 9a).^47^ Another known risk factor for maternal immune activation is obesity^48^ activating microglia via signaling by leptin (Figure 3b).^49^

Interneuron development (Supplementary Figures 9b and c) is controlled by hedgehog, Wnt, nerve growth factor, and transforming growth factor-β signaling^44^ (Figure 3b). Radial glial cells give rise to interneurons via asymmetric division,^50^ affected by disruptions to cell cycle and DNA repair pathways^51^ in Figure 3c. Cellular senescence from DNA damage can occur prematurely with the loss of heparan sulfate^52^ in the extracellular matrix (ECM), consistent with the strong association of GAG metabolism and O-linked glycosylation in Figure 3a. Cell fate decisions by neural progenitors are controlled by p53 (ref. 53) in Figure 3b. Other stress factors include oxidation (Phase 1—functionalization of compounds and Cytochrome P450) as well as dysfunctions to Chaperonin-mediated protein folding and Asn-N-linked glycosylation in Figure 3a. The importance of latter pathways as well as polysialic acids in Golgi was further supported by vesicle-mediated transport in Figure 3c. Interneurons generated migrate to the cortex (Supplementary Figure 9c), regulated by signaling by Rho GTPases and Nuclear signaling by *ErbB4* (Figure 3b).^54^ Neuronal entrance to striatum is avoided by repellant cues including semaphorins and heparan sulfate/CSPGs.^55^ L1 and polysialic acid-modified neural cell adhesion molecule also have important regulatory roles in axon guidance and migration^56, 57^ (L1CAM/NCAM1 interactions in Figure 3a).

These risk factors, resulting in insufficient development of cortical interneurons, likely add up to severe damages upon postnatal synaptic pruning by microglia.^45^ Microglial cells recognize the complement tag C3b, negatively regulated by factor H, which binds GAG chains in ECM^58^ (Supplementary Figure 9e). Neurons are also protected from microglia by polysialic acid-neural cell adhesion molecules, which bind Siglec-11 and downregulate phagocytosis.^59^ The complement activation also produces C3a and C5a, which induce inflammation and recruit phagocytes:^58^ the peptide ligand-binding receptors pathway in Figure 3b describes signaling by G-protein-coupled receptor class A/1 receptors including C3a/C5a receptors. Neuronal phagocytosis is further controlled by the recognition of phosphatidylserine by brain-specific angiogenesis inhibitor 1 (ref. 60) of thrombospondin type 1 repeats-containing protein family, implicating O-glycosylation of thrombospondin type 1 repeats domain-containing protein and glycerophospholipid biosynthesis in Figure 3a. We analyzed pathways highly ranked in SZ and BP similarly and found GAG metabolism, protein glycosylation and cell cycle pathways (Supplementary Figures 10 and 11).

### Analysis of PTSD data

The level of IL association in the two PTSD data sets, ACOD^14, 26^ and MRS,^16^ were similar to SZ/BP results (Supplementary Figure 12). We analyzed gene- and pathway-based SNP groups from the ACOD, MRS and ACOD+MRS data (Supplementary Figure 13): the top-ranked genes were *TBC1D1 (P=*6 × 10^−5^), *HIVEP1* (*P*=9 × 10^−6^) and *PTPRD* (*P*=4 × 10^−5^), respectively, while the distribution of AUCs for pathways was similar to those of SZ and BP in Figure 2. The *PTPRD* gene is third in ranking within the SZ+BD results (Table 1), constituting a replicated finding in PTSD. The highest-ranked pathways were regulation of KIT signaling (AUC=0.555 (0.023), *P*=1 × 10^−4^), peroxisome proliferator-activated receptor α (*PPARA*) activates gene expression (AUC=0.568 (0.027), *P*=2 × 10^−5^) and mitogen-activated protein kinase (*MAPK*) kinase kinase (*MAP3K*)8-dependent MAPK1/3 activation (AUC=0.551 (0.017), *P*=1 × 10^−5^) for the three data sets, respectively. The top two pathways each in MRS and ACOD+MRS exceeded the Bonferroni threshold. We also estimated false discovery rates^61^ using the AUC-*P* regression and found lowest false discovery rate of 0.14, 0.019 and 0.014 for ACOD, MRS and ACOD+MRS data sets, respectively. For the latter two data sets, there were 188 and 591 pathways, respectively, with false discovery rate <0.05 (Supplementary Tables 5 and 6). We compared the rank orders of ACOD+MRS and SZ+BP pathways and found them to be significantly correlated (Kendall's *τ*=0.29, *P*~10^−70^).

### PTSD disease mechanism inferred from pathways

**Figure 4:**
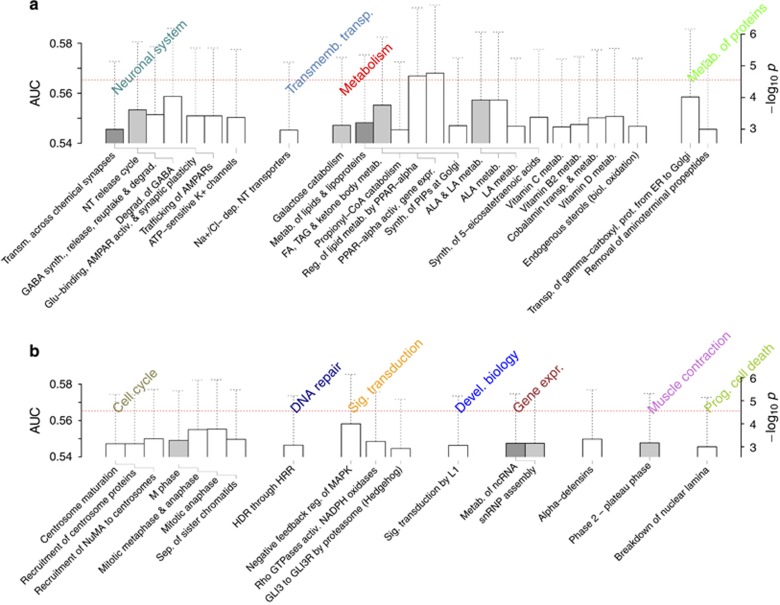
Pathways highly ranked (AUC>0.54 or *P*<2 × 10^−3^) in the PTSD MRS data set (**a** and **b**). Dotted horizontal line represents the Bonferroni threshold. FDR <0.042 for pathways shown. Error bars are 95% confidence interval for AUC. ALA, α-linolenic acid; AUC, area under the curve; carboxyl., carboxylation; CoA, coenzyme A; degrad., degradation; ER, endoplasmic reticulum; FA, fatty acid; FDR, false discovery rate; GABA, γ-aminobutyic acid; GLI3R, GLI family zinc finger 3 repressor; HRR, homologous recombination repair; LA, linoleic acid; MAPK, mitogen-activated protein kinase; MRS, Marine Resilience Study; NADPH, nitotinamide adenine dinucleotide phosphate; ncRNA, noncoding RNA; NT, neurotransmitter; NuMA, nuclear mitotic apparatus; PIPs, phosphatidylinositol phosphates; PPAR, peroxisome proliferator-activated receptor; prog., programmed; prot., protein; PTSD, posttraumatic stress disorder; sep. separation; snRNP, small nuclear ribonucleoprotein; synth., synthesis; TAG, triacylglycerol.

For ACOD, the highly ranked pathways (Supplementary Figure 14) included trafficking of GluR2-containing AMPA receptor, phospholipase C gamma 1 signaling and signaling to extracellular regulated kinase 5 pathway, all of which have key roles in long-term potentiation.^62, 63, 64^ The other pathways of interest were lipid metabolism, transport and signaling pathways, in addition to those involved in neurodevelopment previously observed in SZ/BP: regulation of TP53 activity through association with co-factors, G2 phase (cell cycle) and post-chaperonin tubulin-folding pathway. Many of these pathways were also present in the MRS results (Figure 4), in addition to degradation of GABA, crucial for GABAergic interneurons in controlling fear memory,^65^ negative feedback regulation of MAPK (Figure 4b) central to the Ca^2+^-mediated nuclear activation of transcription in synaptic plasticity^62^ and lipid pathways involving PPAR-α and polyunsaturated fatty acids known to regulate brain functions^66^ (Figure 4a). The neurodevelopmental pathways were similarly represented (Figure 4b), including cell cycle, DNA repair and programmed cell death pathways. The ACOD+MRS results (Supplementary Figure 15) were a consensus of ACOD and MRS with the additional presence of GAG metabolism, biological oxidations and posttranslational protein-modification pathways, all of which previously featured strongly in SZ+BP (Figure 3). Also notable was the phospho-phospholipase A2 (PLA2) pathway of the opioid signaling group (Supplementary Figure 15b).

## Discussion

Our collective inference outcomes demonstrate that association strengths of groups of interacting SNPs are significantly higher than those of non-interacting inference results for psychiatric disorders. Using the relationship between heritability and AUC suggested by Wray *et al.*^67^ and known prevalence,^68^ a SNP group with AUC=0.62 is estimated to account for ~3% of total heritability in liability scale in comparison to ~0.09% for AUC=0.52 typical for IL under the current sample size. Because the rise in association was roughly proportional to aggregate sample sizes under down-sampling (Supplementary Figure 8b), we expect that addition of new samples in meta-analysis inference will continue to enhance the degree of heritability explained by interaction effects. Limitations of our approach, on the other hand, include the reliance on existing databases of pathways and the fact that it is not straightforward to explain the overall association strength of a pathway in terms of genes and individual SNPs contained within a pathway. In addition, the simple assignment of SNPs into genes based on proximity likely fails to capture potentially important regulatory effects of distal noncoding regions.

Evidence suggesting that mast cell activation results from maternal immune activation includes elevated levels of interleukin-6 and tumor necrosis factor during pregnancy, in which neurogenesis and migration occur during its first and second/third trimesters, respectively.^46^ Interleukin-6 and tumor necrosis factor are secreted by IgE-activated mast cells via degranulation,^69^ which influences ECM remodeling pathways.^69^ Mast cells are also known to be activated by the complement system via the action of C3a and C5a^69^ (peptide ligand-binding receptors in Figure 3b). Strong evidence (for example, clinical data indicating disregulation of CSPG-production and ECM abnormalities in SZ patients^70^) suggests the importance of GAG molecules in SZ.^40^ We noted in our results at least three distinct contexts in which GAGs act as negative regulators: preventing premature senescence during neurogenesis, guiding migration and suppressing phagocytosis. The high association of *CSGALNACT1* (Table 1) further indicates that the impaired capacity of sulfated GAG chains to bind ligands is central to disease association. This conclusion is also consistent with the strong association of protein folding, O-linked glycosylation and related pathways in Figure 3a as well as membrane trafficking/transport pathways in Figure 3c. The complement cascade pathway, the target of GAG-mediated negative regulatory action, had AUC=0.591 (0.009), *P*~10^−17^, slightly weaker but comparable to pathways shown in Figure 3. It is also notable that the gene with highest association, *CSMD1* (Figure 2h), encodes a complement inhibitor promoting the degradation of C3b and C4b.^71^ Our results are thus consistent not only with many previous observations of the association of this gene to SZ^32, 33, 34, 35^ and BP,^36, 37, 38, 39^ but also with the recent work on SZ pathogenesis implicating C4 in microglial phagocytosis.^72^

We took advantage of the suspected commonalities^22, 73^ in the etiology of SZ/BP and PTSD to replicate some of our SZ/BP findings in PTSD. The case group sample size of ACOD+MRS is ~20% of SZ+BP (Supplementary Table 1) and the level of PTSD association we found (Supplementary Figure 13) is consistent with the expectation from down-sampling of SZ+BP (Supplementary Figure 8b). Despite the small sample size of the PTSD data sets, *PTPRD* was scored high in both SZ+BP (Figure 2h) and ACOD+MRS results (Supplementary Figure 13c). More importantly, neurodevelopmental pathways highly scored in SZ+BP (Figure 3), including cell cycle, DNA repair, metabolism of carbohydrates and vesicle-mediated transport, were also featured in PTSD (Figure 4 and Supplementary Figure 15). When viewed with our conclusion regarding the pathogenesis mechanism of SZ/BP, this finding suggests a new insight into the genetics of PTSD: insufficient development of inhibitory interneurons, which can lead to SZ/BP if severe, also gives rise to the deficient control of fear responses in the amygdala.

A second prominent feature in the PTSD-associated pathways is synaptic plasticity (Figure 4a, Supplementary Figures 14 and 15a), which is consistent with the fear extinction model. Extinction of conditioned fear, in particular, occurs via long-term potentiation and long-term depression involving NMDA-dependent memory formation, where postsynaptic Ca^2+^ influx leads to AMPA receptor trafficking into postsynaptic density, activation of MAPK, transcription and translation of long-term potentiation-associated proteins, as well as structural remodeling via Rho GTPase-controlled signaling for actin polymerization.^62^ The robust presence of AMPA receptor trafficking pathways and the high associations of MAPK and Rho GTPase pathways (Figure 4b and Supplementary Figure 15b) are consistent with this core component of fear extinction learning. In addition to the deficiency in interneurons owing to impaired neurodevelopment, polymorphisms in the GABAergic signaling system would also affect fear extinction,^62^ as supported by the association of GABA synthesis and degradation pathways in MRS results (Figure 4a). Structural changes also contribute, notably those for ECM,^74^ which is consistent with degradation of ECM in Supplementary Figure 15c.

We also noted high association in fatty acid/phospholipid metabolism and signaling (Figure 4a and Supplementary Figure 15a) known to have important roles in neuronal functions.^66^ Polyunsaturated fatty acids are released from membrane by the action of PLA2, often coupled to neurotransmitter receptors (including NMDAR^75^ and opioid receptors;^76^ phospho-PLA2 pathway in Supplementary Figure 15b). β-Oxidation by cytochrome P450 (Phase 1—functionalization of compounds; Supplementary Figure 15a) into lipid mediators can occur during long-term potentiation^77^ and regulates pain signals^78^ by activating PPAR-α, which acts as an analgesic factor inhibiting nociceptive responses.^79^ The strong association of PPAR-α pathway (Figure 4a) thus suggests that disruptions to the control of pain (such as unconditioned stimulus) may contribute to impaired fear extinction. Consistent with this interpretation, PPAR-α is highly expressed in amygdala and hippocampus^80^ and inhibition of fatty acid amide hydrolase degrading PPAR-α ligands enhances memory acquisition.^81^ It is also notable that the phosho-PLA2 pathway (Supplementary Figure 15b) occurs in the context of opioid signaling, known to have roles in fear extinction^82^ by inhibiting GABAergic antinociceptive pathway.^76^

In conclusion, by testing groups of SNPs for collective association strengths while including non-additive interaction effects, we demonstrated that analyses of moderately sized samples can allow for the discovery of a rich array of pathway groups associated with psychiatric disorders.

## Figures and Tables

**Table 1:** Top 25 genes in high association with SZ+BP under collective inference.

*Rank*	*Gene*	*AUC (95% CI)*	P*-value*	*Known association*^a^
1	*CSMD1*	0.600 (0.009)	5 × 10^−19^	SZ,^32, 33, 34, 35^ BP^36, 37, 38, 39^
2	*CDH13*	0.580 (0.009)	3 × 10^−15^	SZ,^83^ ASD,^84^ ADHD^84^
3	*PTPRD*	0.575 (0.009)	3 × 10^−14^	BP,^37^ ADHD,^85^ OCD^86^
4	*RBFOX1*	0.574 (0.009)	5 × 10^−14^	ASD^87^
5	*WWOX*	0.570 (0.009)	2 × 10^−13^	ARCA^88^
6	*CSGALNACT1*	0.565 (0.009)	3 × 10^−12^	SZ^40^
7	*FHIT*	0.564 (0.009)	3 × 10^−12^	SZ^89^
8	*MAGI2*	0.561 (0.009)	1 × 10^−11^	SZ^90^
9	*LSAMP*	0.559 (0.009)	3 × 10^−11^	BP^8^
10	*CNTN4*	0.558 (0.009)	5 × 10^−11^	SZ^91^
11	*DAB1*	0.556 (0.009)	1 × 10^−10^	SZ,^92^ BP^92^
12	*NPAS3*	0.555 (0.009)	1 × 10^−10^	SZ^93^
13	*LRP1B*	0.554 (0.009)	3 × 10^−10^	SZ^94^
14	*TMEM132D*	0.553 (0.009)	4 × 10^-10^	Anxiety^95^
15	*MACROD2*	0.551 (0.009)	8 × 10^−10^	ASD^96^
16	*ASIC2*	0.550 (0.009)	1 × 10^−9^	Fear in mice^97^
17	*PLCB1*	0.548 (0.009)	3 × 10^−9^	SZ^98^
18	*CTNNA3*	0.547 (0.009)	5 × 10^−9^	SZ^83^
19	*CNTNAP2*	0.547 (0.009)	5 × 10^−9^	SZ^99^, BP^37^
20	*FRMD4A*	0.547 (0.009)	6 × 10^−9^	AD^100^
21	*ASTN2*	0.545 (0.009)	2 × 10^−8^	SZ,^101^ ASD,^102^ ADHD^102^
22	*PCSK5*	0.544 (0.009)	2 × 10^−8^	BP^37^
23	*OPCML*	0.544 (0.009)	2 × 10^−8^	SZ^103^
24	*SORCS2*	0.543 (0.009)	3 × 10^−8^	BP^36^
25	*PTPRT*	0.543 (0.009)	3 × 10^−8^	ASD^104^

Abbreviations: ADHD, attention-deficit hyperactivity disorder; AD, Alzheimer's disease; ARCA, autosomal recessive cerebellar ataxia; ASD, autism spectrum disorder; AUC, area under the curve; BP, bipolar disorder; CI, confidence interval; OCD, obsessive-compulsive disorder; SZ, schizophrenia.

a See Supplementary References.

## Supplementary Material

Supplementary Table 6

Supplementary Software
