# Supplementary material for: Large-scale interaction effects reveal missing heritability in schizophrenia, bipolar disorder and posttraumatic stress disorder
Source: Transl Psychiatry. 2017 Apr 11;7(4):e1089–. doi: 10.1038/tp.2017.61 (PMC5416702; doi:10.1038/tp.2017.61)
Supplement: Supplementary Text, Tables 1 and 2 and Figures [file tp201761x1.pdf]

Large-scale interaction effects reveal missing heritability in schizophrenia, bipolar disorder, and post-traumatic stress disorder: Supplementary Information

HJ Woo, C Yu, K Kumar and J Reifman

Supplementary Text (Meta-analysis algorithm)

We illustrate the basic idea of the discrete discriminant analysis (DDA) meta-analysis using the schizophrenia (SZ) sample containing European American (EA) and African American (AA) sub-populations. The log-likelihood given the data D_k of individual k can be written as

$$L = \sum_k \ln \Pr(D_k) = \sum_{k\in \mathrm{EA}} \ln \Pr(D_k) + \sum_{k\in \mathrm{AA}} \ln \Pr(D_k) = L_{\mathrm{EA}} + L_{\mathrm{AA}}, \tag{1}$$

where the summations are over all individuals in the whole sample, EA, and AA populations, respectively. Each term can be divided further into case and control groups:

$$L_{\mathrm{EA}} = L_{\mathrm{EA},0} + L_{\mathrm{EA},1}, \tag{2}$$

where the subscripts 0 and 1 denote control and case, respectively, and likewise for L_{AA}. Parameter inferences are then performed separately for EA and AA via maximum likelihood such that $L_{\mathrm{EA},0}$ and $L_{\mathrm{EA},1}$ are maximized. In IL, the exact analytic solution is known. For CL, this inference was performed using mean field approximation with regularization parameter $\epsilon \in [0,1]$. Inferences for case and control groups are followed by that of the entire EA sample with $L_{\mathrm{EA,pooled}}$. We then compute

$$q_{\mathrm{EA}} = 2(L_{\mathrm{EA},0} + L_{\mathrm{EA},1} - L_{\mathrm{EA,pooled}}) \tag{3}$$

and likewise for q_{AA} to obtain the statistic

$$q = q_{\mathrm{EA}} + q_{\mathrm{AA}}. \tag{4}$$

The IL p-value was computed from χ^2-distribution with d.f. = 4 (two extra parameters for case-control compared to pooled under genotypic model times two populations). For CL, we performed permutation-based sampling of the null distribution. The parameter sets obtained, e.g., for EA, consist of odds ratios for single SNPs $\beta_i^{(\mathrm{EA})}$ and SNP pairs $\gamma_{ij}^{(\mathrm{EA})}$, where $i, j = 1, \cdots, m$ for m SNPs (for each site i and pair i, j, there are two β_i and four γ_{ij} parameters in genotypic model[9]). We averaged them over sub-groups using[28]

$$\beta_i = \frac{1}{w} \sum_{s=\mathrm{EA,AA}} w_s \beta_i^{(s)} \tag{5a}$$

$$\gamma_{ij} = \frac{1}{w} \sum_{s=\mathrm{EA,AA}} w_s \gamma_{ij}^{(s)}, \tag{5b}$$

where the weight for sub-sample s was taken as

$$w_s = \frac{2}{\sqrt{1/n_0 + 1/n_1}} \tag{6}$$

with n_0 control and n_1 case individuals, and

$$w = \sum_i w_s. \tag{7}$$

Supplementary Table 1. Sample sizes of sub-samples and case-control groups. AA, African American; ACOD, Alcohol, Cocaine, and Opioid-Dependence sample; AS, Asian and others; BP, bipolar disorder; EA, European American; MRS, Marine Resilience Study; SZ, schizophrenia; WT, Wellcome Trust Case Control Study sample.

Sample	Sub-sample	Case	Control	Total
SZ	EA	2 704	2 692[a]	5 396
	AA	1 267	974[a]	2 241
	Total	3 971	3 666	7 637
BP	EA	1 001	1 034[a]	2 035
	AA	347	671[a]	1 018
	WT	1 868	2 938	4 806
	Total	3 216	4 643	7 859
SZ+BP		7 187	8 309[a]	15 496[a]
ACOD	EA	394	1 096	1 490
	AA	424	1 509	1 933
	Total	818	2 605	3 423
MRS	EA	466	3 064	3 530
	AA	33	142	175
	AS	25	98	123
	Total	524	3 304	3 828
ACOD+MRS		1 342	5 909	7 251

[a]Control groups of SZ and BP EA/AA samples share 667 (EA) and 1 009 (AA) individuals, respectively (see Methods).

Supplementary Table 2. Significance test thresholds for gene- and pathway-based SNP groups in different meta-analysis samples.

Sample	Genes		Pathways	
	Number[a]	Bonferroni[b]	Number[a]	Bonferroni[b]
SZ			1 771	2.8×10^{-5}
BP			1 730	2.9×10^{-5}
SZ+BP	17 821	2.8×10^{-6}	1 729	2.9×10^{-5}
ACOD	18 087	2.8×10^{-6}	1 783	2.8×10^{-5}
MRS	18 095	2.8×10^{-6}	1 797	2.8×10^{-5}
ACOD+MRS	18 052	2.8×10^{-6}	1 765	2.8×10^{-5}

[a]Total number of SNP groups with minimum number of 1 (genes) and 20 (pathways) SNPs and valid inference scores (AUC). [b]Bonferroni-corrected nominal threshold for $\alpha = 0.05$.

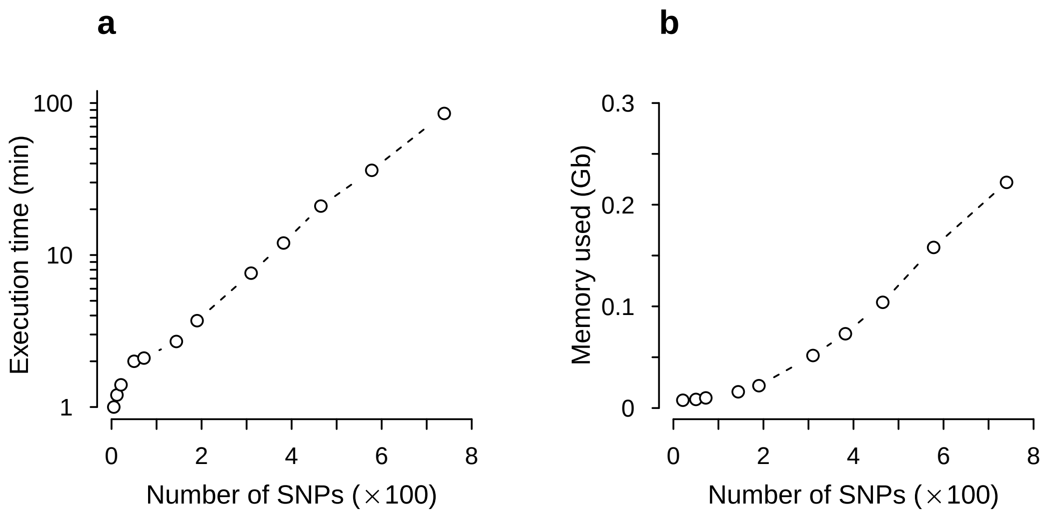

Supplementary Figure 1. Computational costs of collective inference with increasing size of single-nucleotide polymorphism (SNP) sets. (**a**) Execution time in minutes. (**b**) Memory used in gigabytes. For each case, an 8-core Intel Core i7-6700 (3.4GHz) machine was used to run a non-parallel version of mean field inference for 8 values of regularization parameter ϵ.

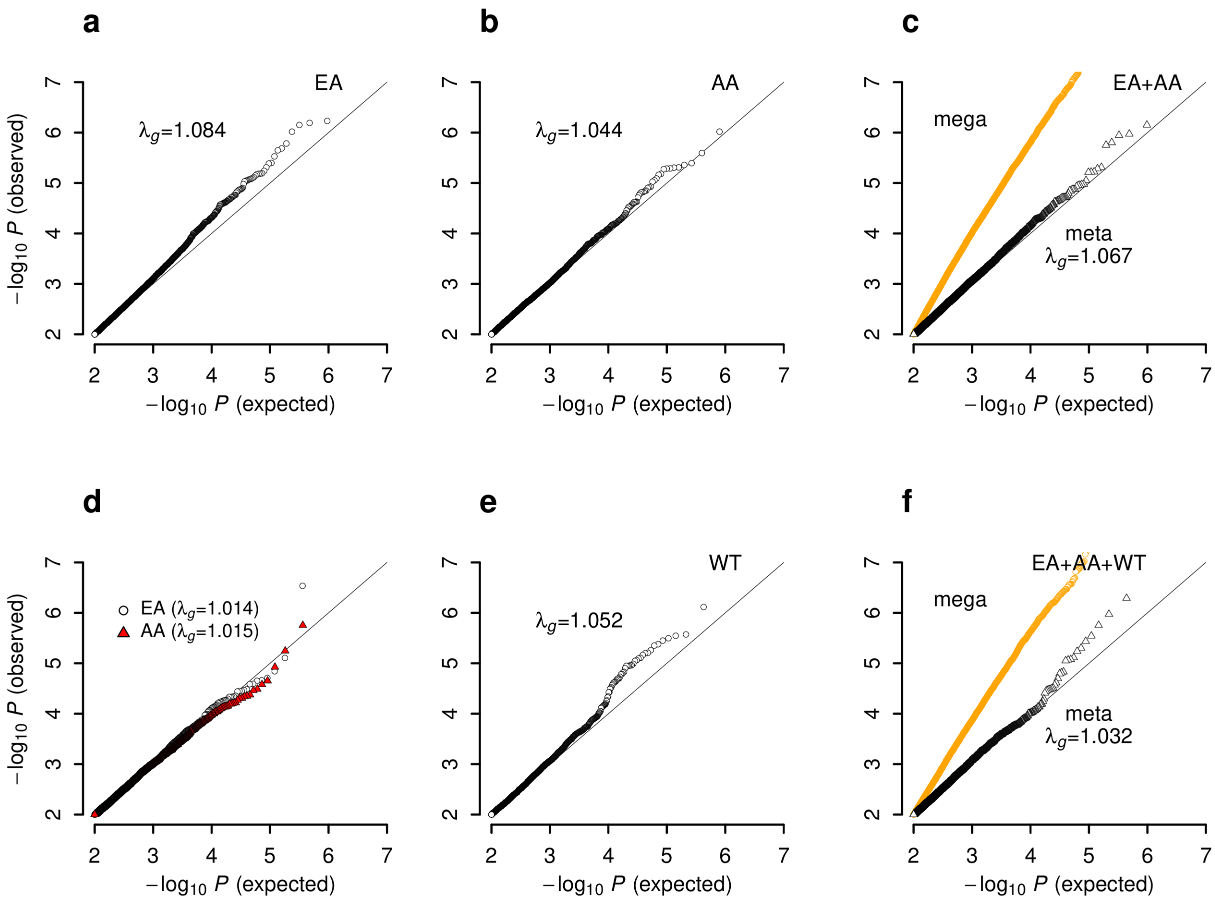

Supplementary Figure 2. Quantile-quantile plots of independent-SNP p-values for SZ/BP datsets. (**a-c**) SZ data with EA, AA sub-samples only, combined data without reference to stratification ('mega'), and meta-analysis ('meta'). λ_g is genomic inflation factor. (**d-f**) BP data with EA, AA, and WT sub-samples.

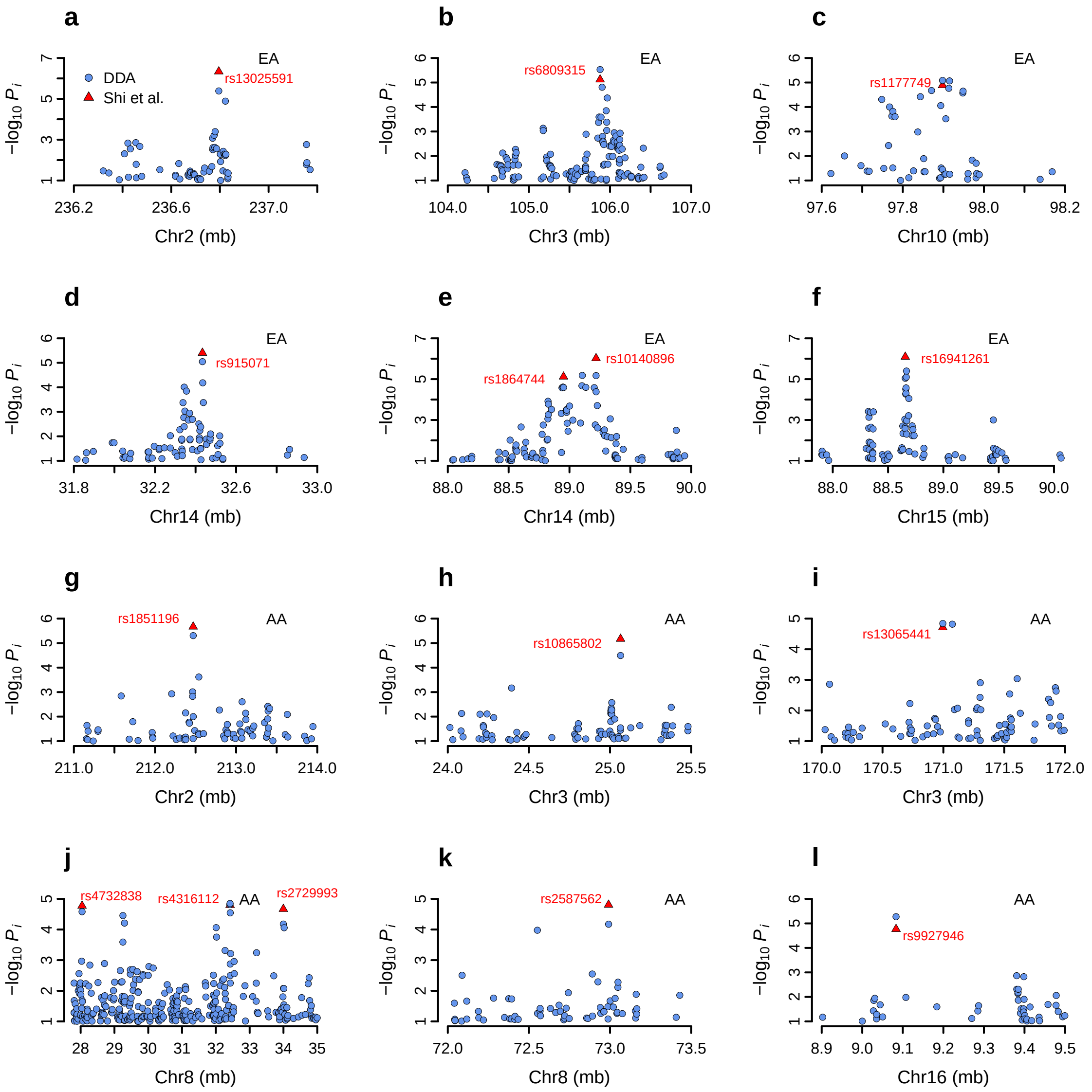

Supplementary Figure 3. Examples of SZ loci with moderate association under independent-SNP assumption. EA and AA sub-samples were used separately for comparison with the summary statistics from Shi et al.,[23] for which SNPs with top association in each loci are shown.

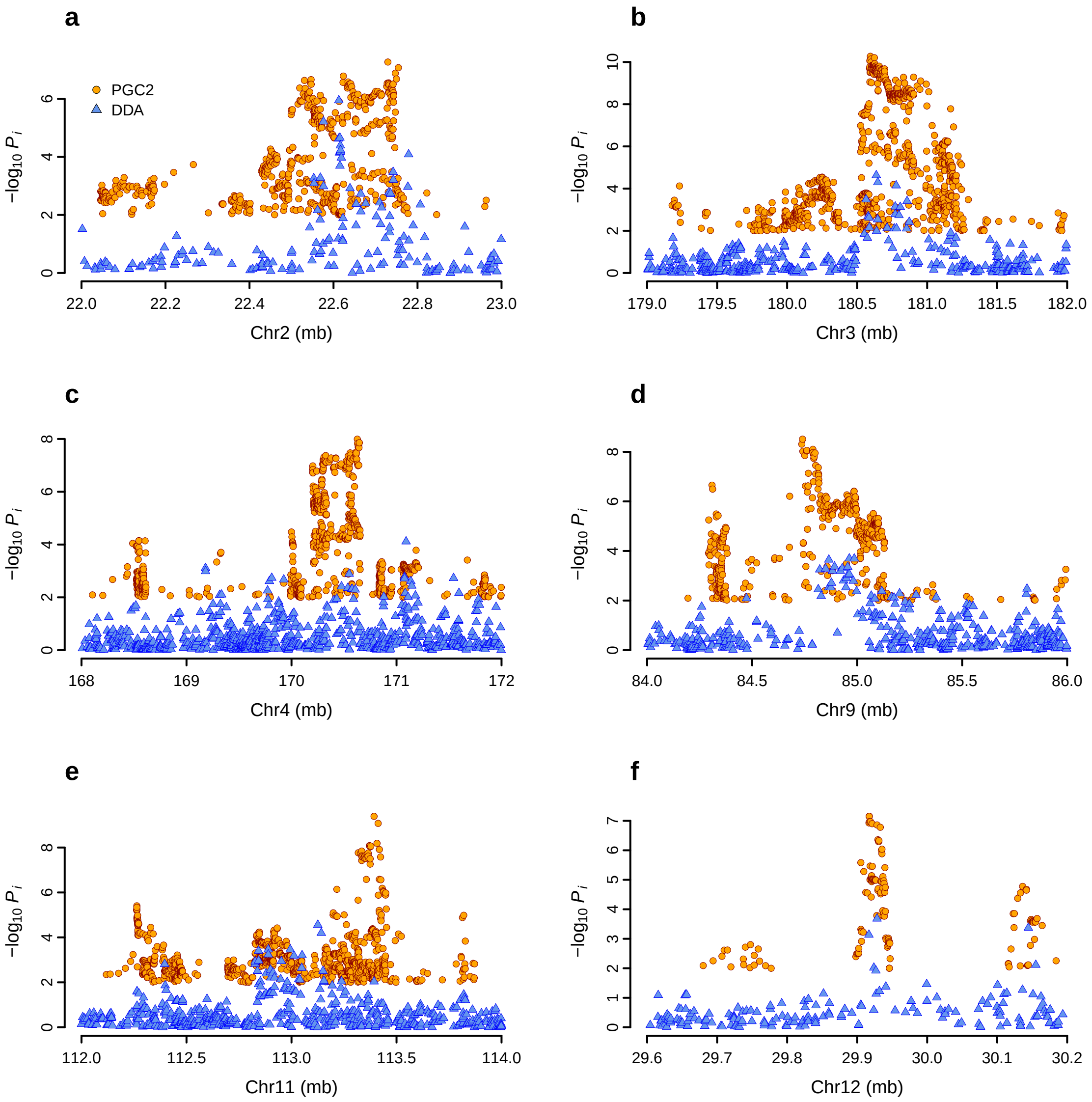

Supplementary Figure 4. Independent-SNP p-value profiles of SZ (EA+AA meta-analysis) compared to the Psychiatric Genomics Consortium 2 (PGC2) summary statistics.[4] Six loci in which the association levels of our datasets and the PGC2 statistics exhibited overlap are shown.

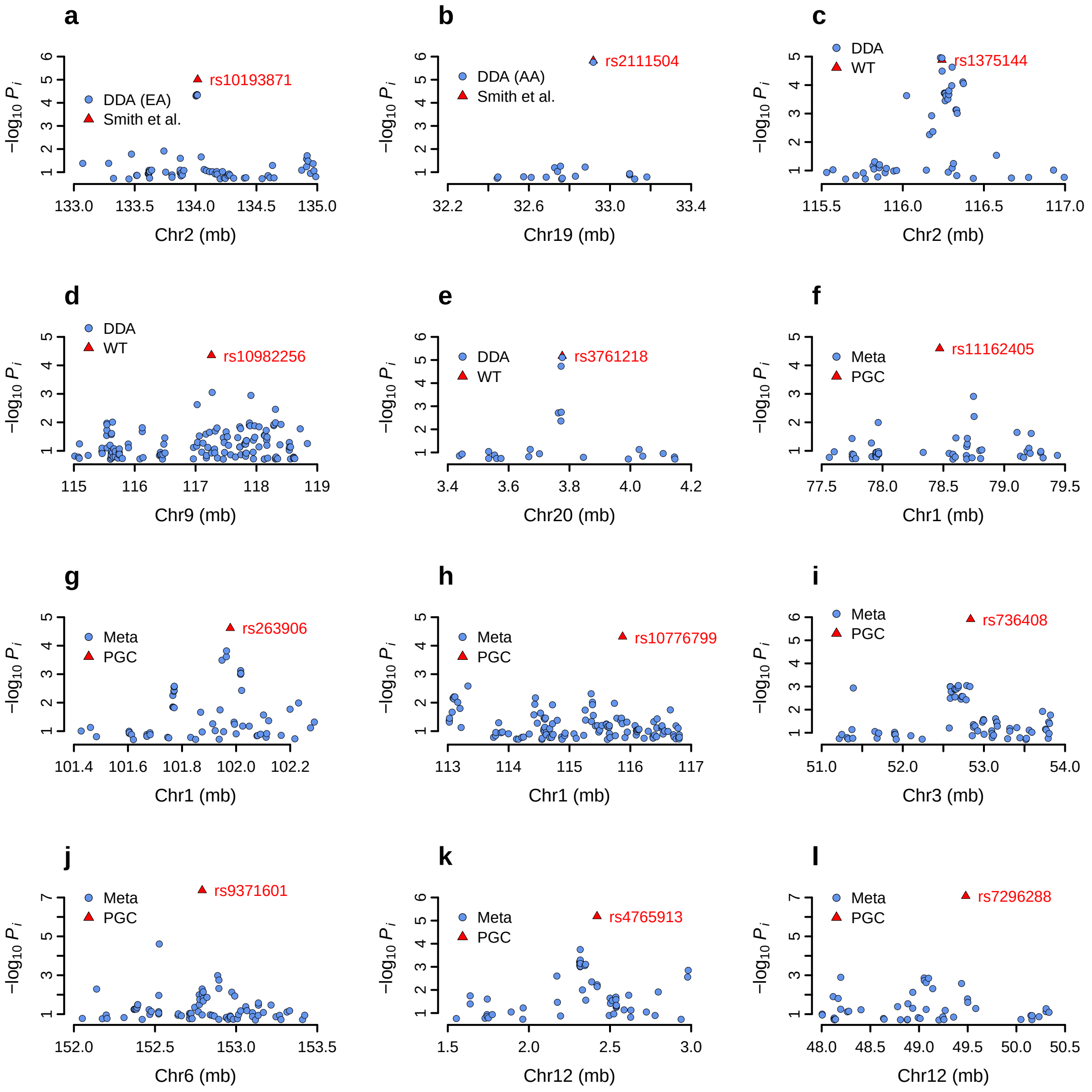

Supplementary Figure 5. Examples of BP loci with moderate association under independent-SNP assumption. (**a,b**) Two loci from EA and AA sub-samples compared with the summary statistics from Smith et al.[24] (**c-e**) Three loci from WT sub-sample compared with original report.[25] (**f-l**) Meta-analysis outcome combining five sub-samples in comparison to the summary statistics from PGC study.[30]

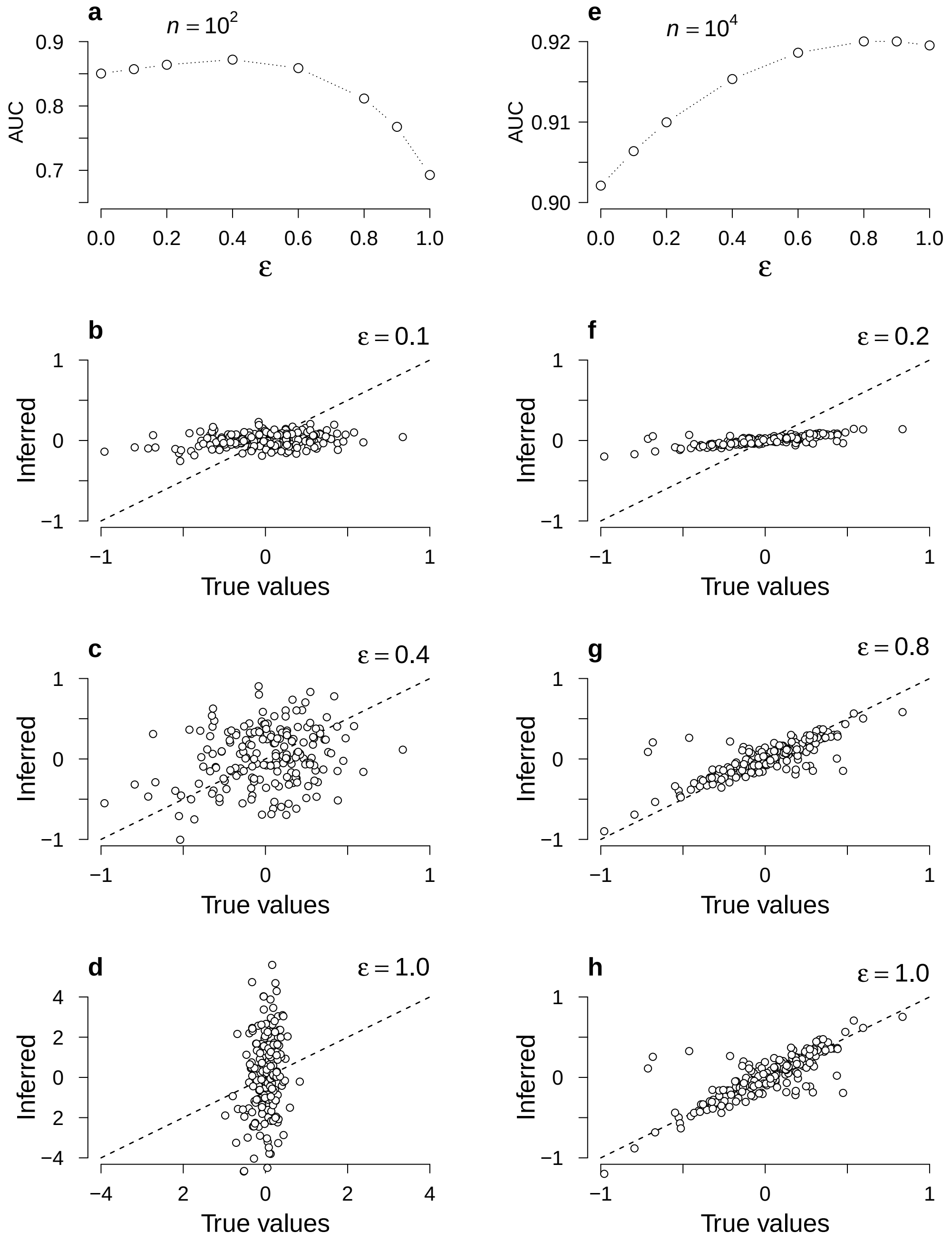

Supplementary Figure 6. Performance of regularized DDA mean field inference on simulated data for $m = 20$ SNPs (dominant model). The model underlying data was specified by the probability distributions of genotypes in case and control groups with parameters for each single-SNP effects (m in total) and interaction terms [$m(m-1)/2$ in total]. These parameters were randomly assigned from normal distributions with mean values of 0 (control) and 0.01 (case) for single-SNP terms, 0 (control) and 0.001 (case) for interactions, respectively, and standard deviations of 0.2. We generated two different datasets of size (case+control) $n = 100$ (**a-d**) and $n = 10^4$ (**e-h**) and performed inferences under different regulalization parameter ϵ values. The limits $\epsilon = 0$ and $\epsilon = 1$ correspond to non-interacting and most strongly interacting cases, respectively. The AUC is maximum at $\epsilon = 0.4$ for $n = 100$ (**a**) and $\epsilon = 0.8$ for $n = 10^4$ (**e**). In **b-d** and **f-h**, we show the comparison of the true interaction parameters and the inferred values (closer to the diagonal line is better; note the change of scale in **d**). With the small sample size of $n = 100$, the inferred parameters were strongly over-fitted near $\epsilon = 1$ (**d**), while the optimal AUC condition correctly identified a good balance at $\epsilon = 0.4$ (**c**). On the other hand, $n = 10^4$ is sufficiently large so that over-fitting was no longer a problem and most interaction parameters were accurately inferred with ϵ close to 1 (**g-h**). Typical conditions using actual data were closer to **a-d**.

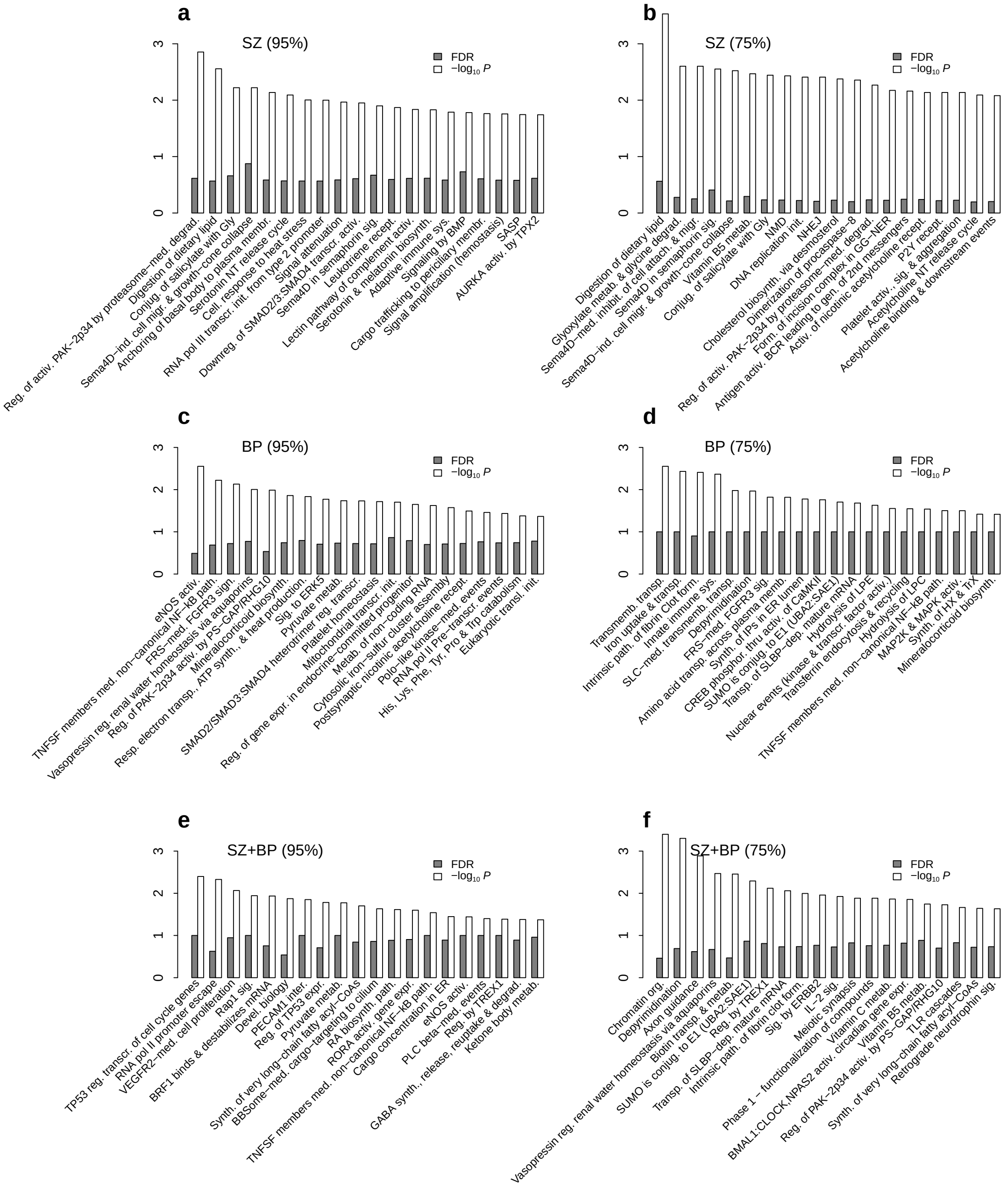

Supplementary Figure 7. Enrichment-based pathway analysis outcome for SZ, BP, and SZ+BP data sets. MAGENTA[31] was used for SZ (**a-b**), BP (**c-d**), and SZ+BP (**e-f**) with results ranked by nominal p-values. The left and right columns show results with 95 and 75 percentile gene score cutoffs, respectively. AURKA, aurora kinase A; BBSome, Bardet-Biedl syndrome proteins; BCR, B cell receptor; BMP, bone morphogenetic protein; conjug., conjugation; CREB, cAMP responsive element binding protein; downreg., downregulation; eNOS, endothelial nitric oxide synthase; ERK, extracellular signal-regulated kinase; FGFR, fibroblast growth factor receptor; FRS, FGFR substrate; GG-NER, global genomic nucleotide excision repair; HX, hepoxilin; induc., induced; IP, inositol phosphate; LPC, lysophosphatidylcholine; LPE, lysophosphatidylethanolamine; migr., migration; NHEJ, non-homologous end-joining; PAK2, p21 (RAC1) activated kinase 2; PLC, phospholipase C; RA, retinoic acid; RHG, Rho GTPase-activating protein; recept., receptor; SASP, senescence-associated secretory phenotype; SLBP, stem-loop binding protein; SLC, solute-carrier; TNFSF, tumor necrosis factor receptor superfamily; TLR, Toll-like receptor; TPX2, microtubule nucleation factor; TrX, trioxilin.

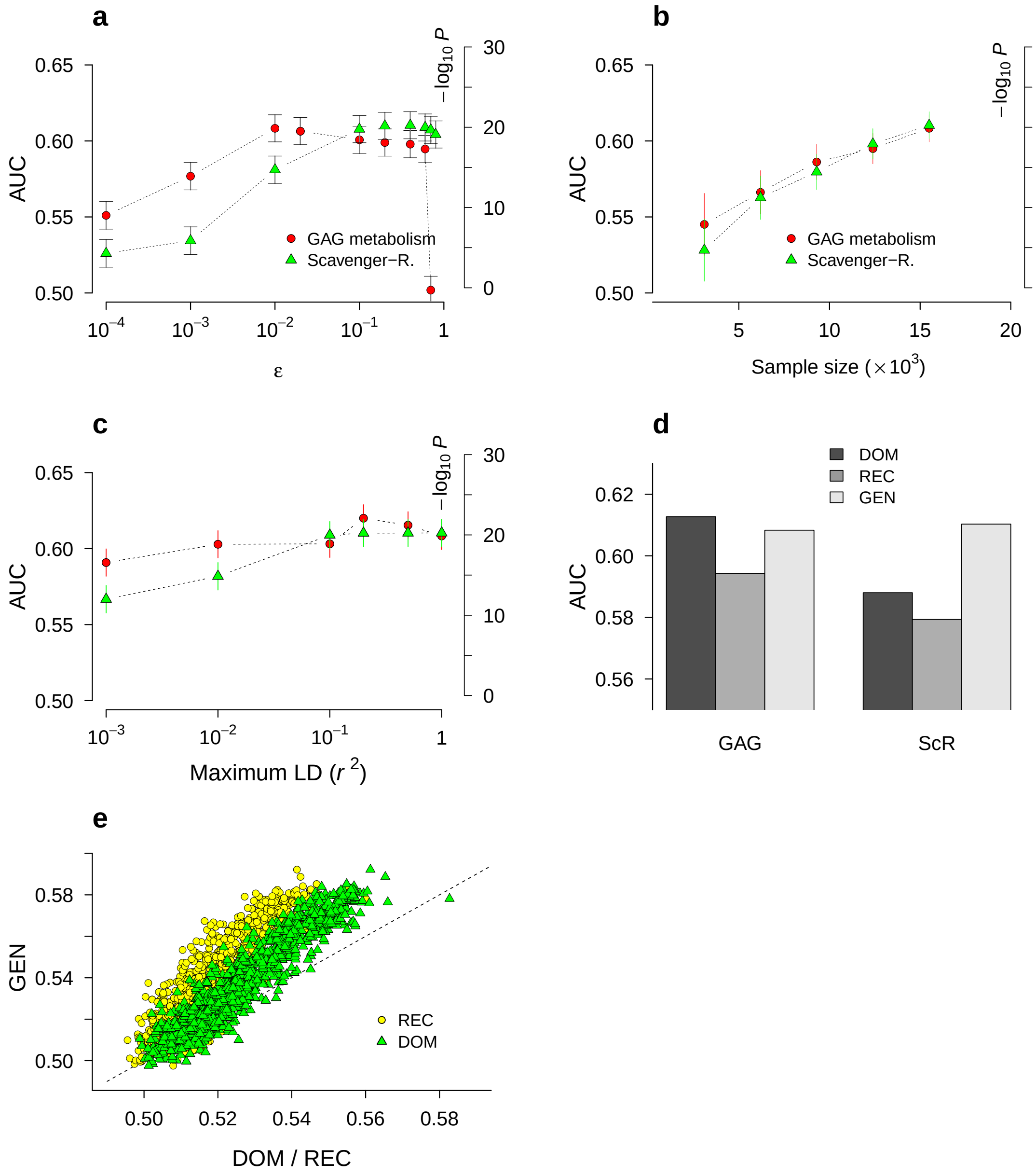

Supplementary Figure 8. Performance of collective inference with varying sample sizes and models. Two pathways, *Glycosaminoglycan (GAG) metabolism* ($m = 2\ 939$ SNPs) and *Binding and uptake of ligands by scavenger receptors* (Scavenger-R./ScR; $m = 974$ SNPs), were used in **a**-**c**. (**a**) Dependence on regularization parameter ϵ. (**b**) Down-sampling of SZ+BP data, where sample size indicates the number of individuals in case and control groups combined. Error bars and vertical lines, 95% c.i. (**c**) Dependence on linkage-disequilibirum (LD)-based pruning. PLINK was used to prune SNPs of two pathways with pairwise LD maximum r^2 indicated (window size 50 and step size 5). The resulting SNP numbers under $r^2 < 0.1$ were 1 242 and 488 for GAG and ScR, respectively. (**d**) Dependence on models [dominant (DOM), recessive (REC) and genotypic (GEN)]. (**e**) Distribution of AUC scores using DOM, REC, and GEN models for a subset of SZ+BP pathways, which indicated the following overall performance sequence: GEN > DOM > REC.

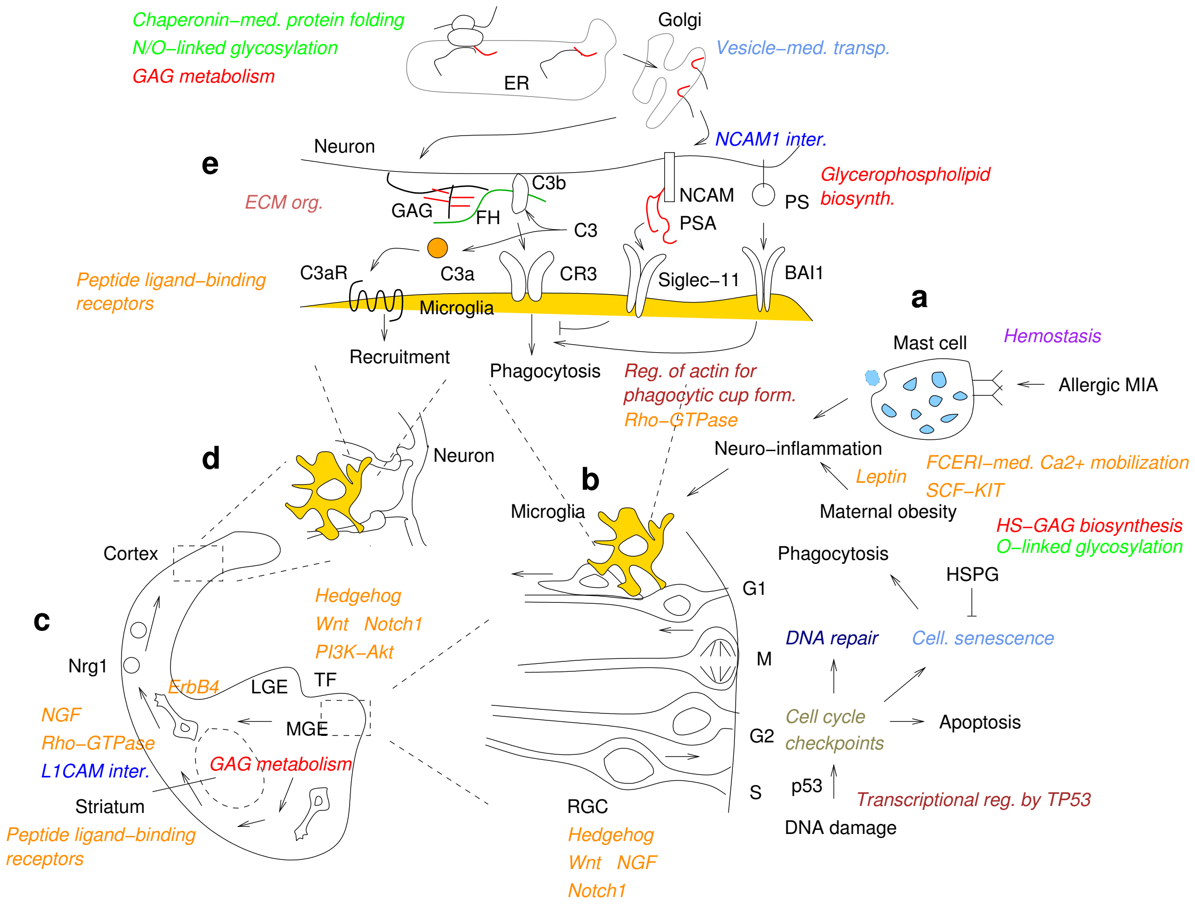

Supplementary Figure 9. Disease mechanism model based on pathways associated with SZ+BP. (**a**) Environmental allergens activate maternal immune response. (**b**) Microglial phagocytosis of neural progenitor cells disrupts neurogenesis of interneurons in the ventral domain of developing telencephalon. Impaired cell cycle, DNA repair, and regulation of cellular senescence contribute to risk. (**c**) Disruptions to tangential migration of neurons requiring guidance cues exacerbate cortical interneuron deficit. (**d**) Synaptic pruning by microglial action on under-developed cortical interneurons triggers disinhibition of excitatory signals. (**e**) In both senescence-induced phagocytosis and synaptic pruning, microglial action occurs via complement activation, negatively regulated by glycoproteins. Proteolytic fragments C3a and C5a further recruit microglia and cause inflammation. Pathways shown in Figure 3 are labeled in italic with the same colors. BAI1, brain-specific angiogenesis inhibitor 1; FH, factor H; LGE, lateral ganglionic eminence; MGE, medial GE; MIA, maternal immune activation; Nrg1, neuregulin-1; PS, phosphatidylserine; PSA, polysialic acid; RGC, radial glial cell; Siglec, sialic acid-binding Ig superfamily lectin.

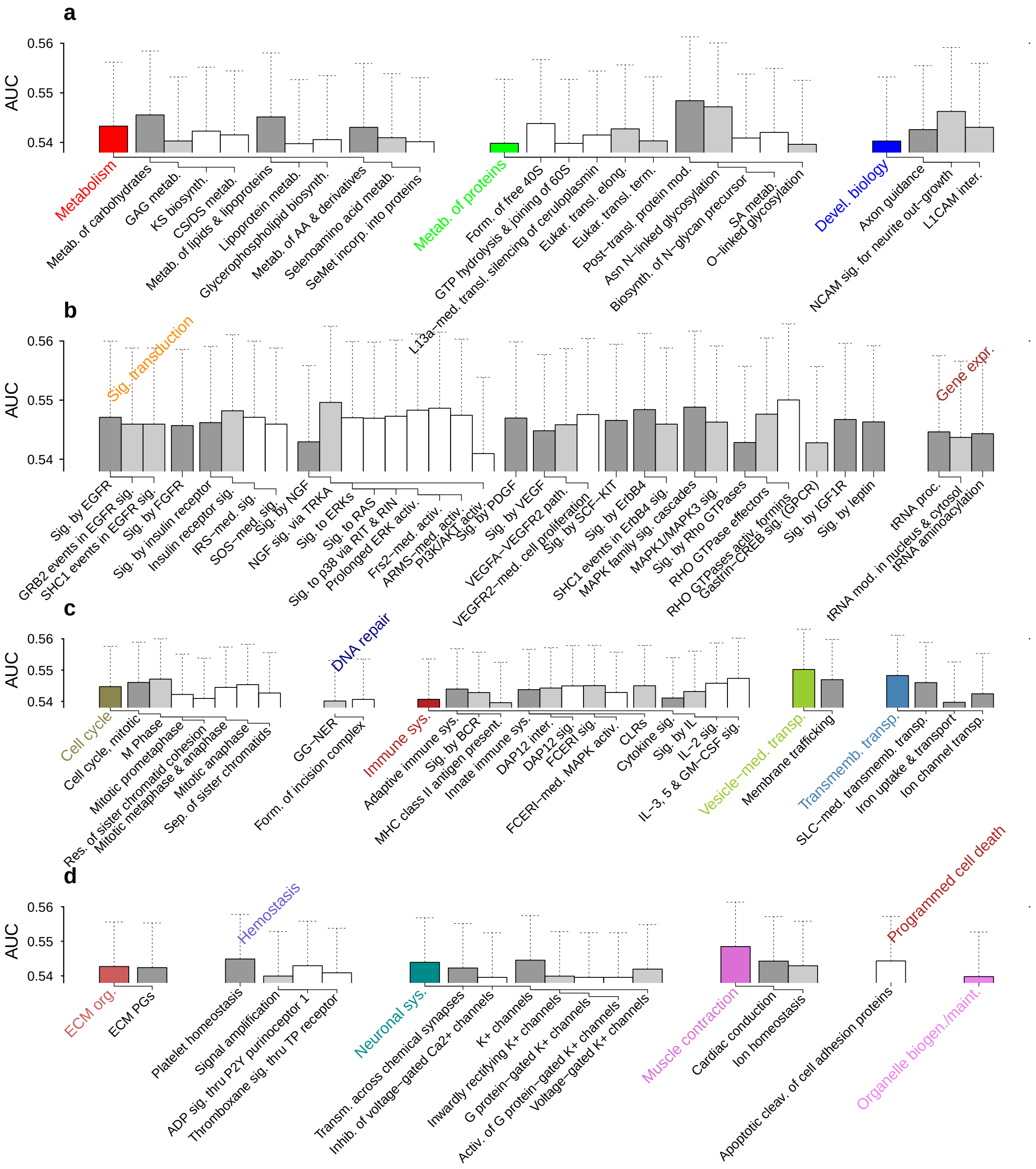

Supplementary Figure 10. Pathways highly associated with SZ under collective inference. (**a-d**) Pathways with AUC > 0.54 in SZ results in Figure 2a. Error bars, 95% c.i. AA, amino acid; ADP, adenosine diphosphate; ARMS, ankyrin-rich membrane spanning; cleav., cleavage; CLR, C-type lectin receptor; DAP12, DNAX activation protein 12; elong., elongation; eukar., eukaryotic; Frs2, fibroblast growth factor receptor substrate 2; incorp., incorporation; inhib., inhibition; IRS, insulin receptor substrate; KS, keratin sulfate; PDGF, platelet-derived growth factor; SeMet, selenomethionine; term., termination; TP, thromboxane receptor; TRKA, tropomyosin receptor kinase A.

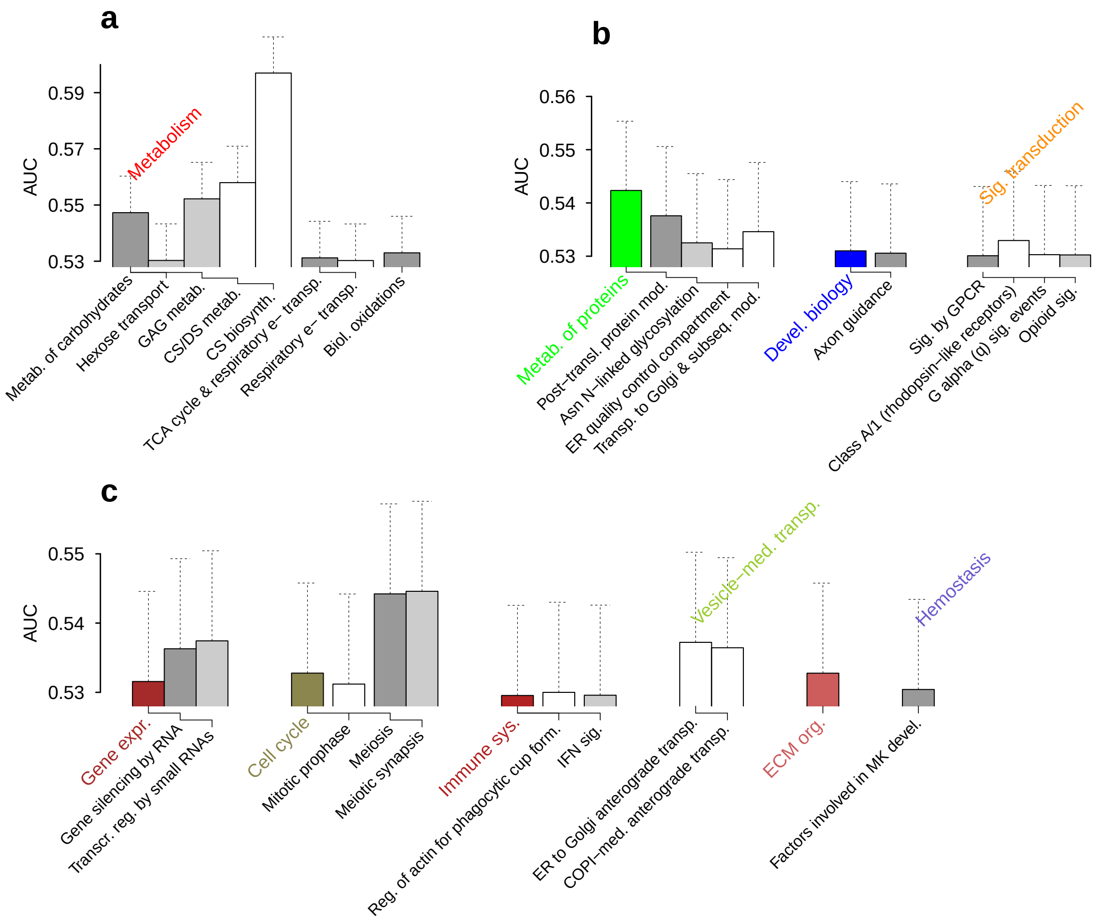

Supplementary Figure 11. Pathways highly associated with BP under collective inference. (**a-c**) Pathways with AUC > 0.53 in BP results in Figure 2c. Error bars, 95% c.i. e-, electron; subseq., subsequent; TCA, the citric acid.

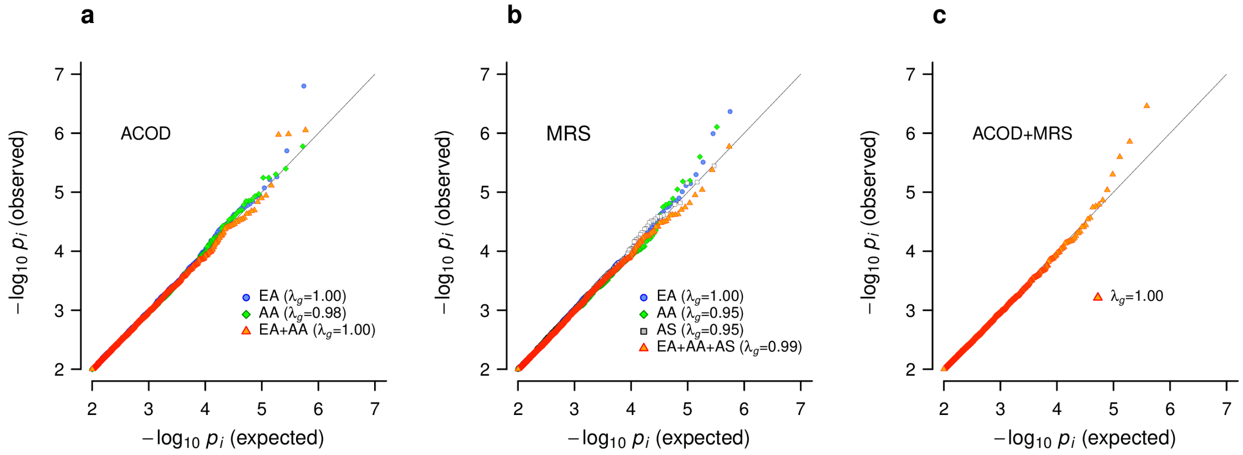

Supplementary Figure 12. Quantile-quantile plots of the post-traumatic stress disorder(PTSD) association strength of SNPs without interaction effects. Three datasets shown are (**a**) ACOD, (**b**) MRS, and (**c**) the combined sample (ACOD+MRS). λ_g is the genomic inflation factor.

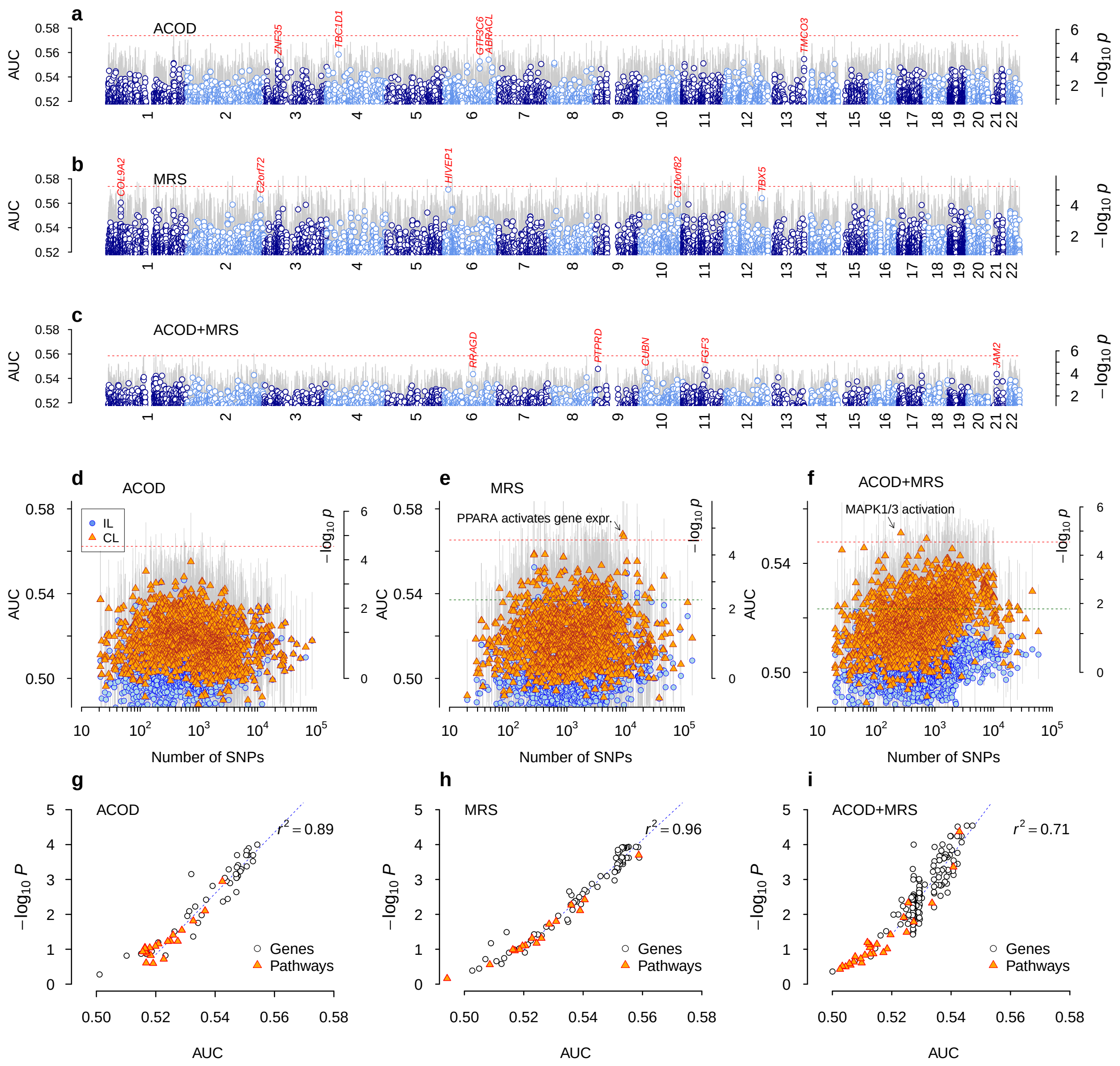

Supplementary Figure 13. Association strengths of gene- and pathway-based SNP groups with PTSD. (**a-c**) Genome-wide distribution of gene-based SNP group associations in three datasets (ACOD, MRS, and ACOD+MRS). Positions in chromosomes indicate the midpoint of the coding region. The top 5 genes are labeled in red in each case. (**d-f**) Distribution of pathway-based SNP group AUC scores with (collective loci; CL) and without (independent loci; IL) interaction effects. Vertical lines represent 95% c.i. of data with interaction effects (CL). (**g-i**) Relationship between AUC and p-values of genes and pathways. Dotted lines represent linear regressions for AUC > 0.52. Axes on the right in **a-f** are based on these regression formulas. Horizontal lines in **a-f** indicate Bonferroni threshold (red) and false discovery rate (FDR) < 0.05 (green). In **d**, the lowest FDR was 0.143. The top pathways exceeding Bonferroni thresholds are labeled in **e** and **f**: *Peroxisome proliferator-activated receptor α (PPARA) activates gene expression*; *MAP3K8-dependent MAPK1/3 activation*.

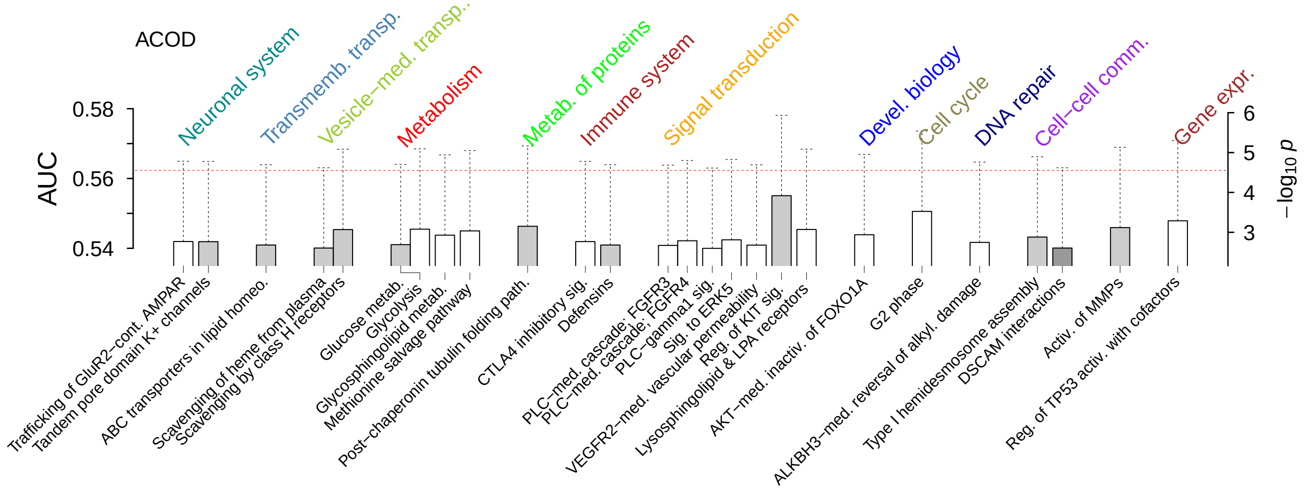

Supplementary Figure 14. Pathways highly ranked (AUC > 0.54 or $P < 2 \times 10^{-3}$) in the ACOD dataset. Dotted horizontal line represents the Bonferroni threshold. Error bars, 95% c.i. ABC, ATP-binding cassette; alkyl., alkylation; AMPAR, α-amino-3-hydroxy-5-methyl-4-isoxazolepropionic acid receptor; cont., containing; CTLA4, cytotoxic T lymphocyte-associated protein 4; DSCAM, Down syndrome cell adhesion molecule, GluR2, metabotropic glutamate receptor 2; homeo., homeostasis; K+, potassium; LPA, lysophosphatidic acid; MMPs, metalloproteinases; path., pathway; VEGFR, vascular endothelial growth factor receptor.

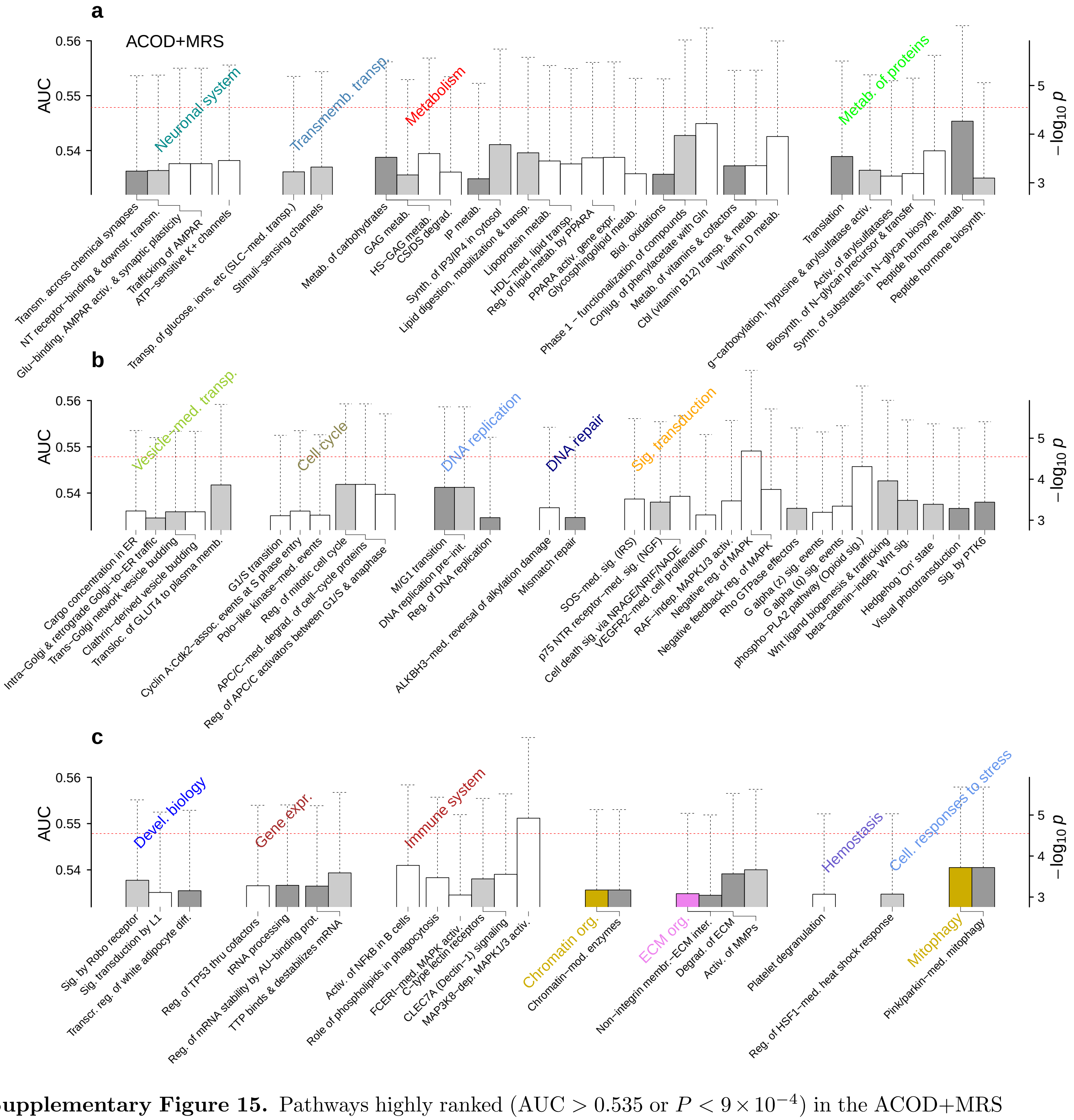

Supplementary Figure 15. Pathways highly ranked (AUC > 0.535 or $P < 9 \times 10^{-4}$) in the ACOD+MRS dataset. Dotted horizontal line represents the Bonferroni threshold. FDR ≤ 0.017 for pathways shown. Error bars, 95% c.i. Cbl, cobalamin; CLEC7A, C-type lectin domain family 7 member A; HDL, high-density lipoprotein; HSF, heat shock factor; NADE, p75 neurotrophin receptor-associated cell death executor; NRAGE, neurotrophin receptor-interacting MAGE homolog; NRIF, nuclear receptor interacting factor; NTR, neurotrophin receptor; PLA2, phospholipase A2; PTK6, protein tyrosine kinase 6; RAF, rapidly accelerated fabrosarcoma; transloc., translocation; TTP, tristetraprolin.

Supplementary References

83. Borglum AD, Demontis D, Grove J, Pallesen J, Hollegaard MV, Pedersen CB *et al.* Genome-wide study of association and interaction with maternal cytomegalovirus infection suggests new schizophrenia loci. *Mol Psychiatry* 2014; **19**(3)**:** 325-333.

84. Redies C, Hertel N, Hubner CA. Cadherins and neuropsychiatric disorders. *Brain Res* 2012; **1470:** 130-144.

85. Elia J, Gai X, Xie HM, Perin JC, Geiger E, Glessner JT *et al.* Rare structural variants found in attention-deficit hyperactivity disorder are preferentially associated with neurodevelopmental genes. *Mol Psychiatry* 2010; **15**(6)**:** 637-646.

86. Mattheisen M, Samuels JF, Wang Y, Greenberg BD, Fyer AJ, McCracken JT *et al.* Genome-wide association study in obsessive-compulsive disorder: results from the OCGAS. *Mol Psychiatry* 2015; **20**(3)**:** 337-344.

87. Fogel BL, Wexler E, Wahnich A, Friedrich T, Vijayendran C, Gao F *et al.* RBFOX1 regulates both splicing and transcriptional networks in human neuronal development. *Hum Mol Genet* 2012; **21**(19)**:** 4171-4186.

88. Mallaret M, Synofzik M, Lee J, Sagum CA, Mahajnah M, Sharkia R *et al.* The tumour suppressor gene WWOX is mutated in autosomal recessive cerebellar ataxia with epilepsy and mental retardation. *Brain* 2014; **137**(Pt 2)**:** 411-419.

89. Raychaudhuri S, Korn JM, McCarroll SA, International Schizophrenia Consortium, Altshuler D, Sklar P *et al.* Accurately assessing the risk of schizophrenia conferred by rare copy-number variation affecting genes with brain function. *PLOS Genet* 2010; **6**(9)**:** e1001097.

90. Walsh T, McClellan JM, McCarthy SE, Addington AM, Pierce SB, Cooper GM *et al.* Rare structural variants disrupt multiple genes in neurodevelopmental pathways in schizophrenia. *Science* 2008; **320**(5875)**:** 539-543.

91. Goes FS, McGrath J, Avramopoulos D, Wolyniec P, Pirooznia M, Ruczinski I *et al.* Genome-wide association study of schizophrenia in Ashkenazi Jews. *Am J Med Genet B Neuropsychiatr Genet* 2015; **168**(8)**:** 649-659.

92. Folsom TD, Fatemi SH. The involvement of Reelin in neurodevelopmental disorders. *Neuropharmacology* 2013; **68:** 122-135.

93. Gonzalez-Penas J, Arrojo M, Paz E, Brenlla J, Paramo M, Costas J. Cumulative role of rare and common putative functional genetic variants at NPAS3 in schizophrenia susceptibility. *Am J Med Genet B Neuropsychiatr Genet* 2015; **168**(7)**:** 528-535.

94. Timms AE, Dorschner MO, Wechsler J, Choi KY, Kirkwood R, Girirajan S *et al.* Support for the N-methyl-D-aspartate receptor hypofunction hypothesis of schizophrenia from exome sequencing in multiplex families. *JAMA Psychiatry* 2013; **70**(6)**:** 582-590.

95. Erhardt A, Czibere L, Roeske D, Lucae S, Unschuld PG, Ripke S *et al.* TMEM132D, a new candidate for anxiety phenotypes: evidence from human and mouse studies. *Mol Psychiatry* 2011; **16**(6)**:** 647-663.

96. Anney R, Klei L, Pinto D, Regan R, Conroy J, Magalhaes TR *et al.* A genome-wide scan for common alleles affecting risk for autism. *Hum Mol Genet* 2010; **19**(20)**:** 4072-4082.

97. Price MP, Gong H, Parsons MG, Kundert JR, Reznikov LR, Bernardinelli L *et al.* Localization and behaviors in null mice suggest that ASIC1 and ASIC2 modulate responses to aversive stimuli. *Genes Brain Behav* 2014; **13**(2)**:** 179-194.

98. McOmish CE, Burrows EL, Howard M, Hannan AJ. PLC-beta1 knockout mice as a model of disrupted cortical development and plasticity: behavioral endophenotypes and dysregulation of RGS4 gene expression. *Hippocampus* 2008; **18**(8)**:** 824-834.

99. Friedman JI, Vrijenhoek T, Markx S, Janssen IM, van der Vliet WA, Faas BH *et al.* CNTNAP2 gene dosage variation is associated with schizophrenia and epilepsy. *Mol Psychiatry* 2008; **13**(3)**:** 261-266.

100. Lambert JC, Grenier-Boley B, Harold D, Zelenika D, Chouraki V, Kamatani Y *et al.* Genome-wide haplotype association study identifies the FRMD4A gene as a risk locus for Alzheimer's disease. *Mol Psychiatry* 2013; **18**(4)**:** 461-470.

101. Vrijenhoek T, Buizer-Voskamp JE, van der Stelt I, Strengman E, Genetic Risk Outcome in Psychosis Consortium, Sabatti C *et al.* Recurrent CNVs disrupt three candidate genes in schizophrenia patients. *Am J Hum Genet* 2008; **83**(4)**:** 504-510.

102. Lionel AC, Tammimies K, Vaags AK, Rosenfeld JA, Ahn JW, Merico D *et al.* Disruption of the ASTN2/TRIM32 locus at 9q33.1 is a risk factor in males for autism spectrum disorders, ADHD and other neurodevelopmental phenotypes. *Hum Mol Genet* 2014; **23**(10)**:** 2752-2768.

103. Moskvina V, Craddock N, Holmans P, Nikolov I, Pahwa JS, Green E *et al.* Gene-wide analyses of genome-wide association data sets: evidence for multiple common risk alleles for schizophrenia and bipolar disorder and for overlap in genetic risk. *Mol Psychiatry* 2009; **14**(3)**:** 252-260.

104. Christian SL, Brune CW, Sudi J, Kumar RA, Liu S, Karamohamed S *et al.* Novel submicroscopic chromosomal abnormalities detected in autism spectrum disorder. *Biol Psychiatry* 2008; **63**(12)**:** 1111-1117.
